# Conditional deficiency of Rho‐associated kinases disrupts endothelial cell junctions and impairs respiratory function in adult mice

**DOI:** 10.1002/2211-5463.13802

**Published:** 2024-04-11

**Authors:** Takahiro Akamine, Takeshi Terabayashi, Takako Sasaki, Riku Hayashi, Ichitaro Abe, Fumihiro Hirayama, Shin‐ichi Nureki, Masahito Ikawa, Haruhiko Miyata, Akinori Tokunaga, Takashi Kobayashi, Katsuhiro Hanada, Dean Thumkeo, Shuh Narumiya, Toshimasa Ishizaki

**Affiliations:** ^1^ Department of Pharmacology, Faculty of Medicine Oita University Yufu Japan; ^2^ Department of Cardiology and Clinical Examination, Faculty of Medicine Oita University Yufu Japan; ^3^ Department of Respiratory Medicine and Infectious Diseases, Faculty of Medicine Oita University Yufu Japan; ^4^ Animal Resource Center for Infectious Diseases Research Institute for Microbial Diseases Suita Japan; ^5^ Department of Experimental Genome Research Research Institute for Microbial Diseases Suita Japan; ^6^ Division of Laboratory Animal Resources, Life Science Research Laboratory University of Fukui Eiheiji‐cho Japan; ^7^ Department of Infectious Disease Control, Faculty of Medicine Oita University Yufu Japan; ^8^ Research Center for GLOBAL and LOCAL Infectious Diseases Oita University Yufu Japan; ^9^ Clinical Engineering Research Center, Faculty of Medicine Oita University Yufu Japan; ^10^ Department of Drug Discovery Medicine Kyoto University Graduate School of Medicine Kyoto Japan

**Keywords:** actin cytoskeleton, cell–cell adhesion, lung function, ROCK, transgenic mice, vascular permeability

## Abstract

The Ras homology (Rho) family of GTPases serves various functions, including promotion of cell migration, adhesion, and transcription, through activation of effector molecule targets. One such pair of effectors, the Rho‐associated coiled‐coil kinases (ROCK1 and ROCK2), induce reorganization of actin cytoskeleton and focal adhesion through substrate phosphorylation. Studies on ROCK knockout mice have confirmed that ROCK proteins are essential for embryonic development, but their physiological functions in adult mice remain unknown. In this study, we aimed to examine the roles of ROCK1 and ROCK2 proteins in normal adult mice. Tamoxifen (TAM)‐inducible ROCK1 and ROCK2 single and double knockout mice (ROCK1^flox/flox^ and/or ROCK2^flox/flox^;Ubc‐CreERT2) were generated and administered a 5‐day course of TAM. No deaths occurred in either of the single knockout strains, whereas all of the ROCK1/ROCK2 double conditional knockout mice (DcKO) had died by Day 11 following the TAM course. DcKO mice exhibited increased lung tissue vascular permeability, thickening of alveolar walls, and a decrease in percutaneous oxygen saturation compared with noninducible ROCK1/ROCK2 double‐floxed control mice. On Day 3 post‐TAM, there was a decrease in phalloidin staining in the lungs in DcKO mice. On Day 5 post‐TAM, immunohistochemical analysis also revealed reduced staining for vascular endothelial (VE)‐cadherin, β‐catenin, and p120‐catenin at cell–cell contact sites in vascular endothelial cells in DcKO mice. Additionally, VE‐cadherin/β‐catenin complexes were decreased in DcKO mice, indicating that ROCK proteins play a crucial role in maintaining lung function by regulating cell–cell adhesion.

## Graphical abstract

Tamoxifen‐induced Rho‐associated coiled‐coil kinase 1 and 2 double knockout (ROCK1/2 DcKO) in adult mice caused the disruption of endothelial barrier in their lungs. In addition, severe pulmonary hemorrhagic plaques were observed. As results, respiratory function was impaired. These findings indicated that ROCK proteins play a crucial role in maintaining lung function by regulating endothelial barrier *in vivo*.

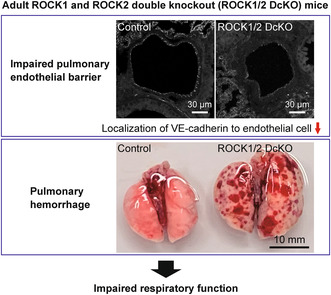

**Abbreviations**
BALF: bronchoalveolar lavage fluid
BP: blood pressure
BUN: blood urea nitrogen
CCL: chemokine ligand
cDNA: complementary DNA
cKO: conditional knockout
DBP: diastolic blood pressure
DcKO: double conditional knockout
DMSO: dimethyl sulfoxide
ELISA: enzyme‐linked immuno‐sorbent assay
ES: embryonic stem
flp: flippase
FRT: flippase‐recombinase target
GSL: *Griffonia simplicifolia* lectin
HR: heart rate
HUVEC: human umbilical vein endothelial cells
i.p.: intraperitoneal
IL: interleukin
IP: immunoprecipitation
LC–MS/MS: liquid chromatography–tandem mass spectrometry
MBP: mean blood pressure
Neo: neomycin
NO: nitric oxide
p120‐CTN: p120‐catenin
PBS: phosphate‐buffered saline
Rho: Ras homology
ROCK: Rho‐associated coiled‐coil kinase
RT‐qPCR: reverse transcription quantitative polymerase chain reaction
SBP: systolic blood pressure
SpO_2_
: percutaneous oxygen saturation
TAM: tamoxifen
TNF‐α: tumor necrosis factor‐α
Ubc: ubiquitin C
VE‐cadherin: vascular endothelial‐cadherin
VEGF: vascular endothelial growth factor
WT: wild‐type
ZO‐1: zonula occludens‐1

The actin cytoskeleton, through remodeling, forms many diverse structures that are highly responsive to the cellular environment and plays a central role in fundamental cellular processes, such as cell migration, polarity, adhesion, and division [1, 2, 3]. In addition to these well‐described functions, structure and remodeling of the actin cytoskeleton have recently been implicated in the maintenance of tissue homeostasis through the regulation of gene transcription and protein degradation [4]. In fact, dysfunctional regulation of the actin cytoskeleton results in a breakdown of organ homeostasis [5, 6, 7].

Upon mechanical or chemical stimulation, the Ras homology (Rho) family of GTPases, which include Rho, Rac, and Cdc42, moderate various steps of actin cytoskeleton remodeling by acting on several downstream effector molecules. Rho‐associated coiled‐coil kinase (ROCK), one of the downstream effectors of Rho, is activated by binding to Rho, and induces reorganization of the actin cytoskeleton through phosphorylation of several substrates [8]. There are two mammalian ROCK isoforms: ROCK1 and ROCK2. Many phosphorylation substrates are commonly shared by ROCK1 and ROCK2, which have highly conserved (about 90% identity) N‐terminal serine/threonine kinase domains [9]. The physiological roles of the ROCKs have been investigated *in vivo* using ROCK transgenic mice and ROCK inhibitors. Both ROCK1 and ROCK2 have demonstrated significant roles in embryonic development [8]. Studies using ROCK2 conventional and conditional knockout (cKO) mice have shown that the deletion of ROCK2 results in specific phenotypes, including placental dysfunction [10], pulmonary vasculopathy [11], cardiac hypertrophy, fibrosis and diastolic dysfunction [12], diabetic nephropathy [13], and anxiety‐like behavior [14]. ROCK1 and ROCK2 double systemic knockout mice on the C57BL/6J background have also been studied; however, these mice die on embryonic Day 3.5. ROCK1^−/−^ROCK2^+/−^ and ROCK1^+/−^ROCK2^−/−^ mice on the C57BL/6J background show a defect of vasculature formation in the yolk sac [15]. In addition, single knockout embryos of either ROCK1 or ROCK2, as well as ROCK1^+/−^ROCK2^+/−^ embryos, show omphalocele and impaired eyelid opening at birth [16]. Thus, ROCK1 and ROCK2 are cooperatively involved in these phenotypes. Owing to the lethality of developmental defects caused by deletion of the ROCK genes, the cooperative functions of ROCK proteins in adult animals have been extensively investigated using nonselective ROCK inhibitors, such as Y‐27632, HA‐1077, and H‐1152. Studies of these compounds have revealed diverse functions of the ROCKs *in vivo* [17]. However, because most of these studies have focused on pathological animal models, the cooperative functions of the ROCK proteins in tissue homeostasis in normal animals are largely unknown.

In this study, we generated a tamoxifen (TAM)‐inducible ROCK1/ROCK2 double knockout mouse using the Cre‐loxP system to elucidate the cooperative roles of the ROCK proteins in normal adult mice. A 5‐day course of TAM led to the death of all ROCK1/ROCK2 double knockout mice by Day 11 post‐treatment. Additionally, hemorrhagic plaques associated with increased vascular permeability were observed in the lungs starting on Day 5 post‐TAM. The localization of vascular endothelial (VE)‐cadherin, p120‐catenin (CTN), and β‐catenin on endothelial cells was reduced in the lungs. There was decreased formation of VE‐cadherin/β‐catenin complexes and decreased expression of each protein. These findings indicated that ROCK1 and ROCK2 might be essential for the maintenance of the pulmonary vascular endothelial barrier.

## Materials and methods

### Reagents

Tamoxifen (TAM; Sigma‐Aldrich, St. Louis, MO, USA) was dissolved in corn oil. Y‐27632 (WAKO, Osaka, Japan) was dissolved in dimethyl sulfoxide (DMSO).

### Ethical considerations in animal procedures

Experimental animals were housed with food and water, which were available *ad libitum*. Animals were treated in accordance with the guidelines stipulated by the Oita University Animal Ethics Committee (approval no. 231101).

### Generation of ROCK1 and ROCK2 double cKO (DcKO) mice

The ROCK1 targeting vector was constructed by flanking exons 3 and 4 of the mouse ROCK1 gene with two loxP sites. One loxP site was inserted 5′ to exon 3 and the other loxP site and the neomycin (Neo) resistance cassette flanked by flippase recombinase target (FRT) sites were inserted 3′ to exon 4. The ROCK2 targeting vector was purchased from EUCOMM (#43842). The targeting vectors for ROCK1 and ROCK2 were electroporated into the embryonic stem (ES) cell lines EGR‐G101 and JM8, respectively. Embryonic stem clones with proper homologous recombination confirmed by polymerase chain reaction (PCR) were microinjected into ICR blastocysts to generate chimeric mice. The chimeric mice were bred with wild‐type (WT) C57BL/6 mice for germline transmission. The resulting offspring were heterozygous for ROCK1^flox/+^ or ROCK2^flox/+^ alleles. These heterozygous animals were then crossed with mice expressing flippase‐recombinase in the germline (FLPeR mice; The Jackson Laboratory, Bar Harbor, ME, USA) to delete the Neo cassette. Double homozygous ROCK1^flox/flox^/ROCK2^flox/flox^ mice were bred with Ubc‐CreERT2 mice (The Jackson Laboratory), in which inducible Cre recombinase is driven by the human ubiquitin C promoter, to generate Ubc‐CreERT2;ROCK1^flox/flox^/ROCK2^flox/flox^ mice. The genotype of the offspring and exon deletion by Cre‐loxP‐mediated recombination were confirmed by PCR amplification using primers listed in Table S1. Primers ROCK1 5′‐loxP (forward) and ROCK1 3′‐loxP (reverse) were used to detect exon deletion of ROCK1. ROCK2 deletion was similarly confirmed using corresponding primer sets. Mice received intraperitoneal (i.p.) administration of TAM once a day for 5 days consecutively. The last day of TAM administration set as Day 0. Mice were euthanized by cervical dislocation, and then organs harvested from mice on the indicated days were used for reverse transcription quantitative PCR (RT‐qPCR), western blotting, immunoprecipitation (IP), and histology, as described below.

### RT‐qPCR analysis

Total RNA from mouse tissues was extracted using the RNeasy Mini Kit (Qiagen, Hilden, Germany). Complementary DNA (cDNA) was synthesized using a ReverTra Ace qPCR RT Kit (Toyobo, Osaka, Japan). RT‐qPCR analysis was performed on diluted cDNA samples using THUNDERBIRD™ SYBR qPCR Mix (Toyobo) with the LightCycler96 System (Roche Diagnostics, Rotkreuz, Switzerland). *β‐actin* was used as an internal control. Primer sequences are listed in Table S2.

### Western blot analysis

Total proteins were extracted and thermally denatured in Laemmli buffer containing mercaptoethanol. The denatured proteins were separated by sodium dodecyl sulfate polyacrylamide gel electrophoresis and transferred to polyvinylidene difluoride membranes. The membranes were incubated with antibodies against ROCK1 [18], ROCK2 (ab71598; Abcam, Cambridge, UK), VE‐cadherin (ab33168; Abcam), β‐catenin (#9562; Cell Signaling Technology, Danvers, MA, USA), p120‐CTN (#59854; Cell Signaling Technology), E‐cadherin (#3195; Cell Signaling Technology), or β‐tubulin (10094‐1‐AP; Proteintech, Rosemont, IL, USA) and then incubated with horseradish peroxidase‐conjugated secondary antibodies. After the membranes were incubated with ECL Prime Western Blotting Detection Reagents (Cytiva, Marlborough, MA, USA), chemiluminescence was detected using an Amersham ImageQuant 800 (Cytiva).

### Hemodynamic parameters

Systolic blood pressure (SBP), mean blood pressure (MBP), diastolic blood pressure (DBP), and heart rate (HR) were measured using the tail cuff method (Softron, Tokyo, Japan), as previously reported [19].

### Measurement of wet/dry lung weight

Lungs were excised from mice on Day 5 post‐TAM. The wet weight of the lungs was measured, then the lungs were incubated at 60 °C for 72 h and the dry weight was measured. The ratio of wet weight to dry weight was calculated.

### Vascular permeability analysis

Vascular permeability was measured by the Evans blue dye test [20]. A 200‐μL solution of 0.5% Evans blue (FUJIFILM Wako Pure Chemical Corporation) in phosphate‐buffered saline (PBS) was injected into the tail vein of each mouse. The lungs, testes, livers, and kidneys were excised 30 min later, and the wet weights of each were measured. The tissues were transferred to 500 μL formamide and then incubated at 60 °C for 24 h to extract Evans blue from the tissues. After centrifugation, the absorbance of the supernatant was measured at 610 nm by a Multiskan SkyHigh Microplate Spectrophotometer (Thermo Fisher Scientific, Waltham, MA, USA) and corrected for the respective weight.

### Histochemical staining

Lungs were excised and fixed with 4% paraformaldehyde (FUJIFILM Wako Pure Chemicals) in PBS (pH 7.4) at 4 °C. Paraffin‐embedded lungs were sectioned at 5 μm thickness and stained with hematoxylin–eosin (Biopathology and Pathology Laboratory, Inc., Oita, Japan). Images were acquired and digitized using a BIOLEVO BZ‐9000 gradient fluorescence microscope (Keyence, Osaka, Japan). Alveolar wall thickness of each specimen was calculated from the mean of the 10 alveoli cross‐sections by Image J (NIH, Bethesta, MD, USA).

### Electron microscopy

Lungs were excised and fixed with 2.5% glutaraldehyde–2% paraformaldehyde in 0.1 m cacodylate buffer (pH 7.4) at 4 °C. Lung specimens were sequentially transferred to the following solutions: 1% osmium tetroxide (TAAB Laboratories Equipment Ltd., Aldermaston, UK) for 1 h, 1% tannic acid (Merck, Darmstadt, Germany) for 1 h, and 1% osmium tetroxide again for 1 h. The samples were dehydrated with ethanol, then lyophilized under vacuum conditions and coated with gold. Images were acquired and digitized using an S‐4800 scanning electron microscope (Hitachi, Tokyo, Japan).

### Percutaneous oxygen saturation (SpO_2_ ) measurement

Monitoring of SpO_2_ was performed using a BM290 pulse oximeter (Biomachinery Co., Ltd., Chiba, Japan). A Common Recognition and Identification Platform‐type sensor was attached to one hind leg of the mice under minimal isoflurane anesthesia. SpO_2_ was measured immediately after awakening.

### Bronchoalveolar lavage

Bronchoalveolar lavage was carried out as previously reported [21]. Briefly, 1 mL PBS was administered to mice by intratracheal instillation, and then collected. This process was repeated five times. The total number of cells in the bronchoalveolar lavage fluid (BALF) was counted using a hemocytometer. Bronchoalveolar lavage fluid was smeared on glass slides, and then stained with May–Grünwald–Giemsa. The number of macrophages or neutrophils was calculated from the percentage of 300 cells. Interleukin (IL)‐6 and chemokine ligand (CCL)2 concentrations in BALF were measured using Mouse IL‐6 and MCP‐1 ELISA Kits (Proteintech), respectively, following the manufacturer's protocol.

### Measurement of histamine content by liquid chromatography–tandem mass spectrometry (LC–MS/MS)

Lungs were excised on Day 5 following the TAM course and rinsed in PBS. Wet weight of each lung was measured. Samples were frozen and crushed in 100 μL 10 mm HCl, to which 400 μL 10 μm 2‐morpholonoethnesulfonic acid (DOJINDO, Kumamoto, Japan) solution in MeOH was added; incubated at room temperature for 30 min; then centrifuged at 12 000 ***g*** at 4 °C for 15 min. A 400 μL aliquot of each supernatant was transferred into a 0.5‐mL tube with a 10‐kDa cutoff filter and centrifuged at 12 000 ***g*** at 4 °C for 1 h. Histamine content of the purified solution was measured by an LC–MS 8040 (Shimadzu, Kyoto, Japan) using the Shimadzu Metabolites Method Package. A Discovery HS F5‐3 column (inner diameter, 2.1 mm; length, 150 mm; particle size, 3 μm) from Sigma‐Aldrich was used to separate compounds. The mobile phase gradient (A = 0.1% formic acid in water and B = 0.1% formic acid in acetonitrile) was as follows: 0.0–2.0 min, 0% B; 2.0–5.0 min, 0–25% B; 5.0–11.0 min, 25–35% B; 11.0–15.0 min, 35–95% B; 15.0–20.0 min 95% B; 20.0–25.0 min 0% B. Flow rate was 0.25 mL·min^−1^, and column temperature was 40 °C. The primary transitions monitored were *m*/*z* 112.10 > *m*/*z* 95.05 for histamine in positive ion mode, and *m*/*z* 194.00 > *m*/*z* 80.15 for the 2‐morpholonoethnesulfonic acid in negative ion mode. The ratios of histamine peak area to 2‐morpholonoethnesulfonic acid peak area were calculated and corrected for the respective weight.

### Immunohistochemistry and phalloidin staining

Lungs were fixed with 4% paraformaldehyde in PBS (pH 7.4) at 4 °C overnight. Fixed lungs were transferred to 10% sucrose in PBS overnight, and then 20% sucrose in PBS overnight. Lungs embedded with OCT compound (Sakura‐Finetek, Tokyo, Japan) were sectioned at 8 μm thickness, then blocked in PBS containing 1% bovine serum albumin and 0.1% Triton X‐100 at 4 °C for 1 h. For phalloidin staining, the sections were incubated with Alexa Fluor™ 488 phalloidin (Thermo Fisher Scientific) at 4 °C for 1 h. For immunohistochemistry, the sections were incubated with antibodies against VE‐cadherin (1 : 100, ab282277; Abcam), β‐catenin (1 : 200, #9562S; CST, Danvers, MA, USA), p120‐CTN (1 : 200, #59854 T; CST), zonula occludens‐1 (ZO‐1; 1 : 100, sc‐33 725, Santa Cruz Biotechnology, Dallas, TX, USA) at 4 °C overnight. These antibodies were detected with a fluorescence‐conjugated secondary antibody (1 : 1000, Alexa 488; Jackson ImmunoResearch, Laboratories, West Grove, PA, USA). *Griffonia simplicifolia* lectin (GSL) I‐Isolectin B4 conjugated with DyLight 594 (Vector Laboratories, Burlingame, CA, USA) was used to detect vascular endothelial cells in accordance with the manufacturer's instructions. Sections were mounted using the TrueVIEW Autofluorescence Quenching Kit from Vector Laboratories. Images were acquired and digitized on an FV3000 confocal scanning laser microscope (Olympus, Tokyo, Japan) or an SP8 confocal scanning laser microscope (Leica, Wetzlar, Germany). Fluorescence intensity was calculated by the software attached to the FV3000.

### IP

Lungs or livers were harvested from TAM‐treated ROCK1/ROCK2 double‐floxed mice (control) and ROCK1/2 DcKO mice on Day 5 or Day 7, respectively. Cultured mouse vascular endothelial‐like (eEND2) cells [22] were seeded on plastic dishes with or without Matrigel (40%) coating, and then cultured for 24 h in Endothelial Cell Basal Medium 2 containing SupplementPack (PromoCell, Heidelberg, Germany) at 37 °C and 5% CO_2_ in a humidified incubator. The cells were treated with either DMSO or 15 μm Y‐27632 for 1 h. The cells seeded on Matrigel were incubated with Cell Recovery Solution (Corning, Corning, NY, USA) according to the manufacturer's instructions, and then collected by centrifugation at 1000 ***g***. The tissues and cells were lysed with IP buffer containing 25 mM Tris–HCl (pH 7.5), 100 mM NaCl, 1% NP‐40, and proteinase inhibitors. After centrifugation, the tissue and cell extracts were incubated with anti‐β‐catenin antibody (ab32572; Abcam) conjugated to Dynabeads Protein A (Invitrogen, Waltham, MA, USA). The beads were washed three times with IP buffer, and precipitated proteins were extracted with Laemmli sample buffer and then analyzed by western blotting.

### Statistical analysis

Data are expressed as means ± standard deviation (SD). All statistical analyses were carried out using the two‐tailed Student's *t*‐test.

## Results

### Cooperation between ROCK1 and ROCK2 is essential to maintain physiological homeostasis

**Fig. 1:**
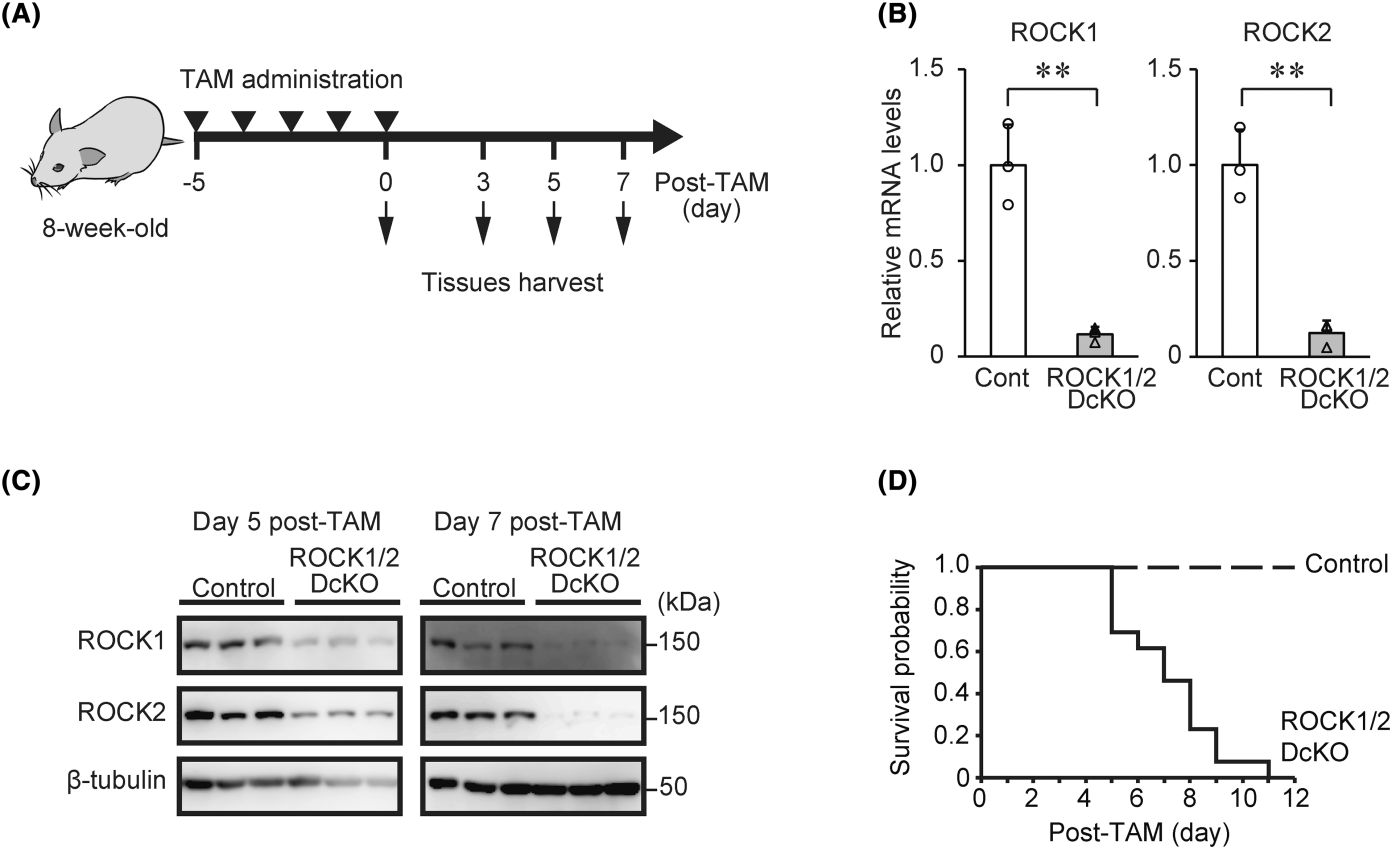
Generation of Rho‐associated coiled‐coil kinase 1 and Rho‐associated coiled‐coil kinase 2 double conditional knockout (ROCK1/2 DcKO) mice. (A) Protocol for induction of Cre recombinase activity in 8‐week‐old mice administered tamoxifen (TAM; 2 mg day^−1^, intraperitoneal) once daily for 5 consecutive days. Mice were sacrificed on Days 0, 3, 5, or 7 post‐TAM and organ tissues were harvested for reverse transcription quantitative polymerase chain reaction (RT‐qPCR), western blotting, immunoprecipitation, and histology. (B) RT‐qPCR analysis of Rho‐associated coiled‐coil kinase 1 (ROCK1) and Rho‐associated coiled‐coil kinase 2 (ROCK2) mRNA expression in the lungs of control and ROCK1/2 DcKO mice on Day 5 post‐TAM. Data are shown as the means ± SD of fold changes relative to control mice (*n* = 3); **P < 0.01 vs. control mice, as determined by Student's *t*‐test. (C) Western blot analysis of lung lysates from control and ROCK1/2 DcKO mice on Days 5 and 7 post‐TAM using the indicated antibodies. Each group contains protein obtained from three different individuals. (D) Kaplan–Meier survival plots of control and ROCK1/2 DcKO mice post‐TAM. Survival probabilities were calculated using control and ROCK1/2 DcKO mice (*n* = 13).

We generated floxed ROCK1 (ROCK1^flox/flox^) and ROCK2 (ROCK2^flox/flox^) mice using targeting vectors with loxP sites flanking exons 3–4 of the ROCK1 gene and exon 3 of the ROCK2 gene, respectively (Fig. S1). These single‐floxed mice were crossed to generate ROCK1/ROCK2 double‐floxed mice, then crossed with Ubc‐CreERT2 mice to generate the following: Ubc‐CreERT2;ROCK1^flox/flox^ (ROCK1 cKO), Ubc‐CreERT2;ROCK2^flox/flox^ (ROCK2 cKO), and Ubc‐CreERT2;ROCK1/ROCK2 (ROCK1/2 DcKO) mice. ROCK1/ROCK2 double‐floxed mice were used as control mice in this study. Mice were administered TAM (2 mg day^−1^, i.p.) once daily for five consecutive days (Fig. 1A). To evaluate the efficacy of the ROCK1 and ROCK2 DcKO, we performed RT‐qPCR and western blot analyses of multiple organ tissues from ROCK1/2 DcKO mice. On Day 5 following the completion of the TAM course, RT‐qPCR analysis showed that mRNA levels of ROCK1 and ROCK2 in the lungs were significantly decreased in ROCK1/2 DcKO mice compared with those in control mice (ROCK1: 0.12 ± 0.04‐fold, *P* < 0.01; ROCK2: 0.12 ± 0.07‐fold, *P* < 0.01; Fig. 1B). These levels were also reduced by > 85% in the testes, livers, and kidneys of ROCK1/2 DcKO mice (Fig. S2A). Western blot analysis showed that the protein levels of ROCK1 and ROCK2 were decreased in the lungs and livers of ROCK1/2 DcKO mice compared to those of control mice on Days 5 and 7 post‐TAM (Fig. 1C and data not shown). The decrease in these proteins was greater on Day 7 than on Day 5. These results demonstrated the efficiency of the cKO of both ROCK1 and ROCK2 in ROCK1/2 DcKO mice following TAM treatment. Next, we examined the phenotypic characteristics of ROCK1/2 DcKO mice. Measurement of the survival rate post‐TAM revealed that ROCK1/2 DcKO mice started dying on Day 5 following the completion of the TAM course, with all mice dead by Day 11 (Fig. 1D). In contrast, ROCK1 cKO and ROCK2 cKO mice did not die by Day 11 post‐TAM administration (data not shown). As previous studies have shown, ROCK signaling plays a role in the regulation of blood pressure (BP) [23]. Therefore, we examined the BP and HR of mice deficient in either ROCK1 or ROCK2, or both. No statistically significant differences in SBP, MBP, DBP, or HR were observed among ROCK1 cKO, ROCK2 cKO, and control mice on Day 5. However, on Day 5 following the TAM course, ROCK1/2 DcKO mice had significant reductions in SBP, MBP, and DBP, but not HR, compared to control mice (Table 1). These results indicated that ROCK1 and ROCK2 play complementary roles in BP regulation.

**Table 1:** Rho‐associated coiled‐coil kinase 1 (ROCK1) and Rho‐associated coiled‐coil kinase 2 (ROCK2) cooperatively contribute to blood pressure (BP) regulation. Measurements of heart rate (HR), systolic blood pressure (SBP), mean blood pressure (MBP), and diastolic blood pressure (DBP) in control (*n* = 14), ROCK1 conditional knockout (ROCK1 cKO; *n* = 12), ROCK2 conditional knockout (ROCK2 cKO; n = 8), and ROCK1/2 double conditional knockout (ROCK1/2 DcKO; *n* = 8) mice on day 5 following the course of tamoxifen (TAM) treatment. Data are shown as means ± SD; **P < 0.01 vs. control mice, as determined by Student's *t*‐test.

	Control	ROCK1 cKO	ROCK2 cKO	ROCK1/2 DcKO
HR (beat·min^−1^)	616.3 ± 46.2	617.7 ± 59.5	618.1 ± 54.4	583.7 ± 44.7
SBP (mmHg)	104.5 ± 19.2	105.0 ± 18.4	101.0 ± 10.5	84.1 ± 9.7**
MBP (mmHg)	81.4 ± 15.5	68.7 ± 11.7	67.0 ± 11.6	58.3 ± 8.2**
DBP (mmHg)	68.2 ± 14.9	50.8 ± 9.8	50.8 ± 12.9	43.8 ± 9.1**

### ROCKs are required to maintain pulmonary vascular permeability

**Fig. 2:**
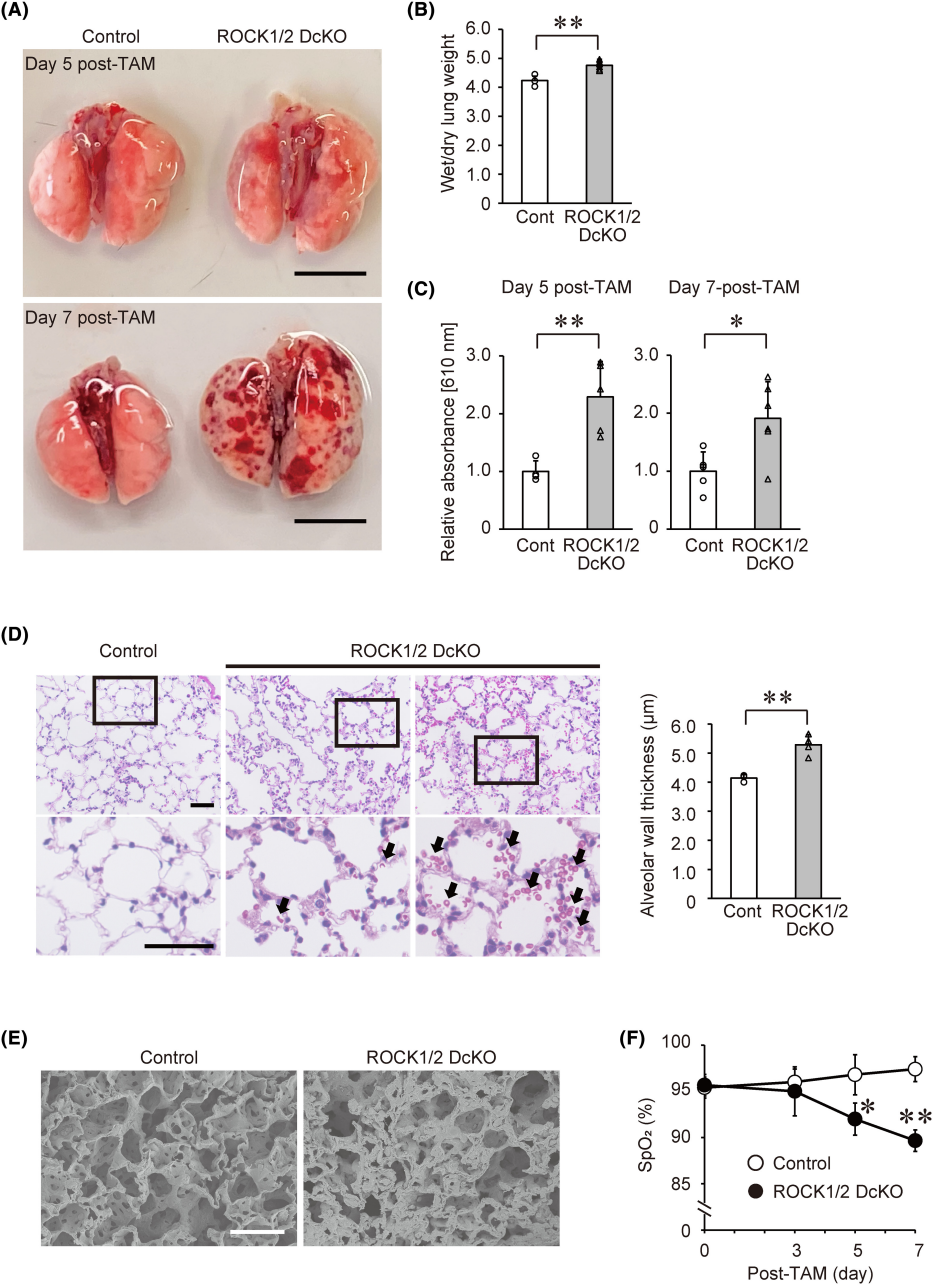
Pulmonary hemorrhage and impaired respiratory function in Rho‐associated coiled‐coil kinase 1 and Rho‐associated coiled‐coil kinase 2 double conditional knockout (ROCK1/2 DcKO) mice. (A) Representative photographs of lungs from control and ROCK1/2 DcKO mice on Days 5 (top panel) and 7 (bottom panel) following the course of tamoxifen (TAM) treatment. Scale bar: 10 mm. (B) Wet/dry weight ratios of lungs excised from control (*n* = 3) and ROCK1/2 DcKO (*n* = 6) mice on Day 5 post‐TAM. Ratios are shown as means ± SD; **P < 0.01 vs. control mice, as determined by Student's *t*‐test. (C) Relative absorbance of Evans blue dye extracted from lungs of control and ROCK1/2 DcKO mice excised 30 min post‐dye injection on days 5 (*n* = 4 for control mice and *n* = 5 for ROCK1/2 DcKO mice; left panel) and 7 (*n* = 5 for control mice and *n* = 6 for ROCK1/2 DcKO mice; right panel) post‐TAM and measured at 610 nm. Absorbance values were corrected for each lung weight and shown as means ± SD of fold changes relative to control mice; *P < 0.05, **P < 0.01 vs. control mice, as determined by Student's *t*‐test. (D) Representative photomicrographs of hematoxylin–eosin stained lung sections in control (left panels) and ROCK1/2 DcKO (middle and right panels) mice on Day 5 post‐TAM. The upper right panel shows a lung section with severe hemorrhages. The lower panels contain enlarged images of the boxed areas in the upper panels. Red blood cells leaking into alveoli (arrows) were observed in ROCK1/2 DcKO mice. Scale bar: 50 μm. Quantification of alveolar wall thickness in hematoxylin–eosin stained lung sections in control and ROCK1/2 DcKO mice on Day 5 post‐TAM. Thicknesses are shown as means ± SD (*n* = 4); **P < 0.01 vs. control mice, as determined by Student's *t*‐test. (E) Representative photomicrographs of lungs in control (left) and ROCK1/2 DcKO (right) mice on Day 5 post‐TAM obtained by scanning electron microscopy. Scale bar: 100 μm. (F) Percutaneous oxygen saturation (SpO_2_) levels in control (*n* = 3) and ROCK1/2 DcKO (*n* = 5) mice measured over the course of 7 days post‐TAM. Data are shown as means ± SD; *P < 0.05, **P < 0.01 vs. control mice at the corresponding time points, as determined by Student's *t*‐test.

**Fig. 3:**
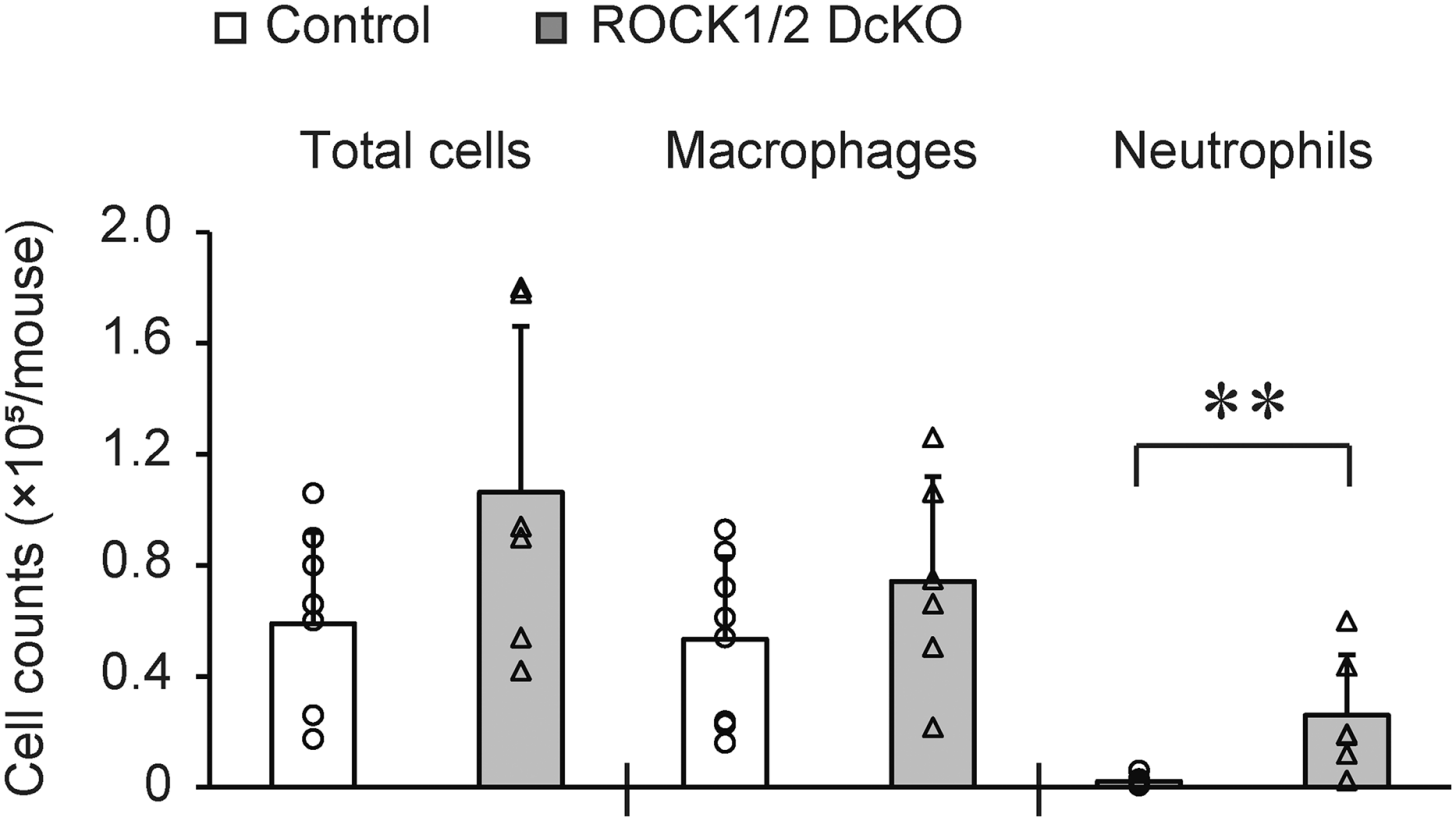
Migration of neutrophils into alveoli in Rho‐associated coiled‐coil kinase 1 and Rho‐associated coiled‐coil kinase 2 double conditional knockout (ROCK1/2 DcKO) mice. Numbers of total cells, macrophages, and neutrophils in the bronchoalveolar lavage fluid (BALF) of control (*n* = 8) and ROCK1/2 DcKO (*n* = 6) mice on Day 5 following the course of tamoxifen (TAM). Cell counts are shown as means ± SD of the total cells counted with a hemocytometer, and the numbers of macrophages or neutrophils calculated from the percentage of 300 cells; **P < 0.01 vs. control mice, as determined by Student's *t*‐test.

ROCK1/2 DcKO mice exhibited a decrease in BP and began dying from Day 5 post‐TAM onwards, and therefore, we observed the appearance of organs from these mice on Days 5 and 7 (Fig. 2A and Fig. S2B). On Day 7 following the completion of the TAM course in ROCK1/2 DcKO mice, we observed hemorrhages throughout the lungs and increased lung size compared with control mice. On Day 5, by contrast, most ROCK1/2 DcKO mice did not have lung hemorrhages on gross examination; a minority of mice from this group exhibited limited numbers of small hemorrhages. Therefore, the ratio of wet to dry lung weight was measured on Day 5 post‐TAM (Fig. 2B). These ratios in control and ROCK1/2 DcKO mice were 4.23 ± 0.21 and 4.75 ± 0.15, respectively, showing a significant increase in ROCK1/2 DcKO mice (*P* < 0.05). Next, we examined whether the deficiency in both ROCK1 and ROCK2 affected vascular permeability (Fig. 2C). The Evans blue dye test showed significantly higher dye absorbance in the lungs of ROCK1/2 DcKO mice on Days 5 (2.29 ± 0.61‐fold, *P* < 0.01) and 7 (1.91 ± 0.63‐fold, *P* < 0.05) compared to that in control mice. A similar increase in absorbance was observed in the testes on Day 7, but not in the livers or kidneys (Fig. S2C). Furthermore, histological analysis showed that erythrocytes leaked into the alveoli of ROCK1/2 DcKO mice on Day 5 post‐TAM (Fig. 2D). In some ROCK1/2 DcKO mice, leakage of erythrocytes was found, even in the absence of apparent hemorrhage of the lungs (Fig. 2D, middle panels). Furthermore, the alveolar wall thicknesses were measured on Day 5 post‐TAM. These values in control and ROCK1/2 DcKO mice were 4.14 ± 0.21 μm and 5.28 ± 0.35 μm, respectively, showing a significant increase in ROCK1/2 DcKO mice (*P* < 0.01). This thickening of alveolar walls was confirmed by scanning electron microscopy (Fig. 2E). Additionally, in ROCK1/2 DcKO mice, there was a tendency toward an increased total number of alveolar cells and a significantly increased number of neutrophils compared with control mice (Fig. 3). Because of the increased vascular permeability in ROCK1/2 DcKO mice, we assessed respiratory function by measuring SpO_2_ (Fig. 2F). Control mice maintained SpO_2_ > 95% throughout the measurement period. In contrast, on Day 5 post‐TAM, the SpO_2_ of ROCK1/2 DcKO mice (92.0 ± 1.7%) was significantly lower than that of control mice (*P* < 0.05); on Day 7, the SpO_2_ of ROCK1/2 DcKO mice deteriorated further (89.7 ± 1.2%, *P* < 0.01).

**Fig. 4:**
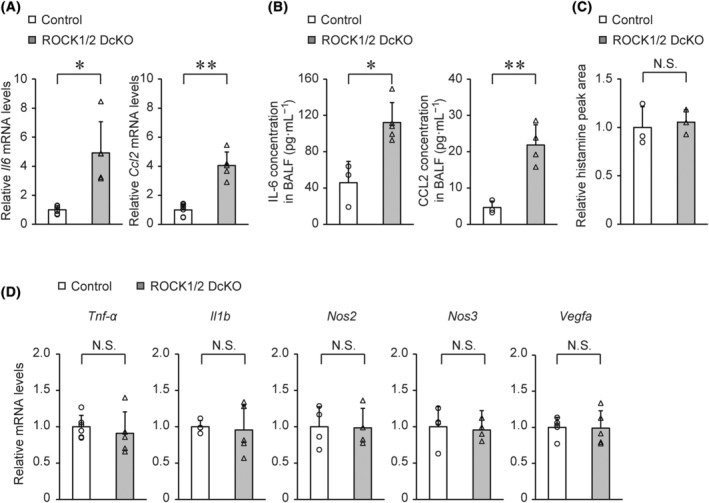
Increased levels of interleukin‐6 (IL‐6) and chemokine ligand 2 (CCL2) in the lungs of Rho‐associated coiled‐coil kinase 1 and Rho‐associated coiled‐coil kinase 2 double conditional knockout (ROCK1/2 DcKO) mice. (A) Reverse transcription quantitative polymerase chain reaction (RT‐qPCR) measurement of *Il6* and *Ccl2* mRNA expression levels in lungs of control (*n* = 6) and ROCK1/2 DcKO (*n* = 5) mice on Day 5 following the tamoxifen (TAM) course. Data are shown as the means ± SD of fold changes relative to control mice; *P < 0.05, **P < 0.01 vs. control mice, as determined by Student's *t*‐test. (B) Enzyme‐linked immuno‐sorbent assay (ELISA) measurements of IL‐6 and CCL2 protein concentrations in bronchoalveolar lavage fluid (BALF) from control (*n* = 3) and ROCK1/2 DcKO (*n* = 5) mice on Day 5 post‐TAM. Data are shown as means ± SD; *P < 0.05, **P < 0.01 vs. control mice, as determined by Student's *t*‐test. (C) Liquid chromatography–tandem mass spectrometry (LC–MS/MS) measurements of histamine content in control and ROCK1/2 DcKO mice on Day 5 post‐TAM. The histamine peak areas were corrected for each internal standard peak area. Data are shown as means ± SD of fold changes relative to control mice (*n* = 3), and were analyzed by Student's *t*‐test. (D) RT‐qPCR analysis of mRNA expression of tumor necrosis factor‐α (*Tnf‐α*; *n* = 6 for control mice and *n* = 5 for ROCK1/2 DcKO mice), interleukin‐1β (*Il1b*; *n* = 4 for control mice and *n* = 5 for ROCK1/2 DcKO mice), nitric oxide related genes (*Nos2*, *Nos3*; *n* = 4 per group), and vascular endothelial growth factor (*Vegfa*; *n* = 5 per group) in lungs of control and ROCK1/2 DcKO mice on Day 5 post‐TAM. Data are shown as means ± SD of fold changes relative to control mice and were analyzed by Student's *t*‐test.

Increased vascular permeability is involved in the induction of many inflammation‐related factors, including tumor necrosis factor (TNF)‐α, IL‐1β, histamine, nitric oxide (NO), and vascular endothelial growth factor (VEGF) [24, 25]. Levels of proinflammatory cytokines IL‐6 and CCL2 were elevated in ROCK1/2 DcKO mice compared to those in control mice on Day 5 post‐TAM (Fig. 4A,B). However, there was no significant between‐group differences in the lung levels of substances that increase vascular permeability directly, such as histamine (Fig. 4C), TNF‐α, IL‐1β, NO‐related genes, and VEGF (Fig. 4D) on Day 5 post‐TAM. Consequently, despite the observation that ROCK1/ROCK2 deficiency triggered lung inflammation, we speculated that mechanisms other than inflammation may be responsible for the increased vascular permeability in ROCK1/2 DcKO mice.

### Effects of ROCK deficiency on phalloidin staining and VE‐cadherin complex localization

**Fig. 5:**
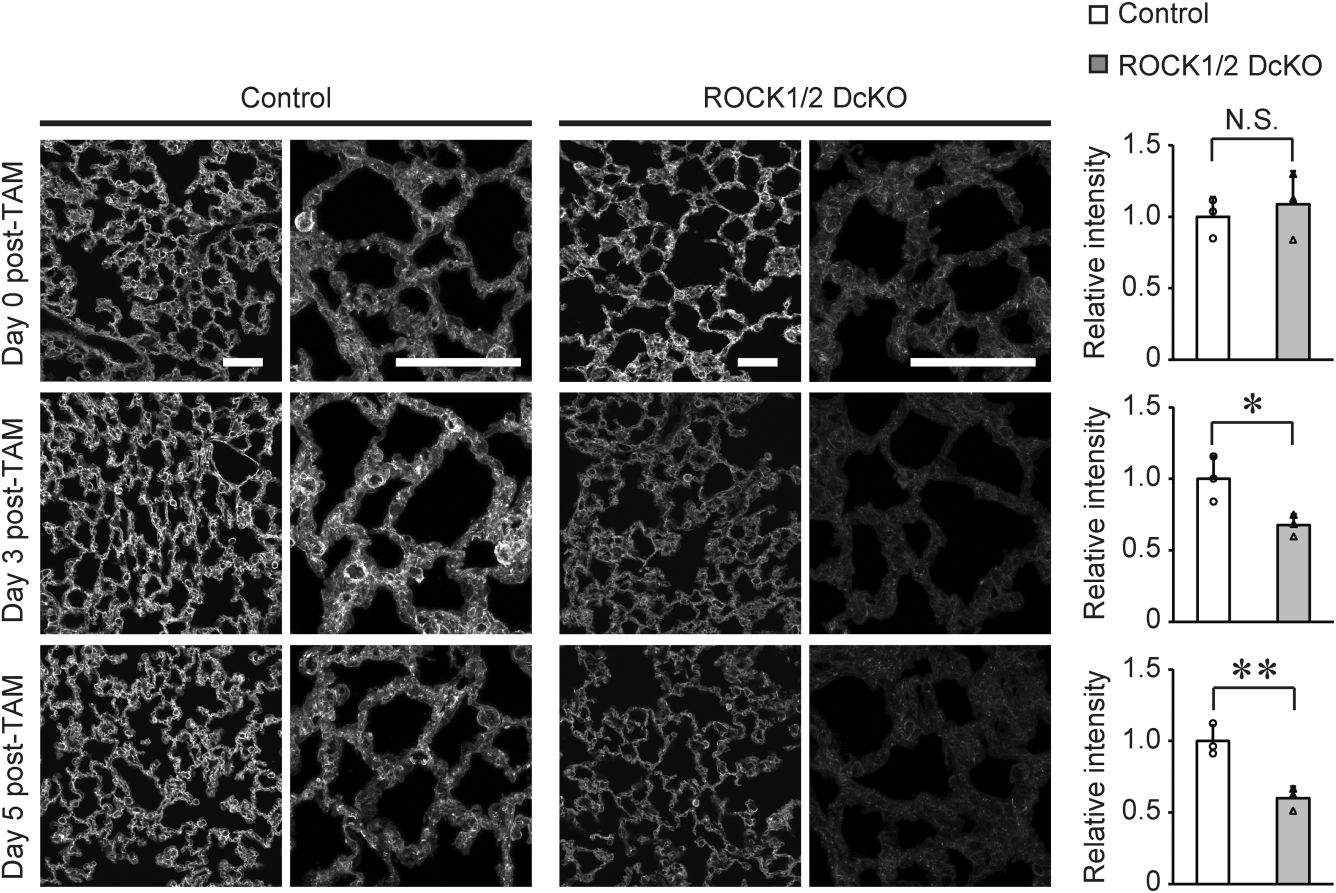
Phalloidin intensity is reduced in the lungs of Rho‐associated coiled‐coil kinase 1 and Rho‐associated coiled‐coil kinase 2 double conditional knockout (ROCK1/2 DcKO) mice. Representative images of phalloidin staining in the lungs of control mice and ROCK1/2 DcKO mice on Days 0, 3, and 5 following the course of tamoxifen (TAM) treatment. Low (left panels) and high (right panels) magnification images are shown. High magnification images of entire tissue were acquired every 0.7 μm, and the overlay images are shown. Scale bars: 50 μm. Two lines were drawn in each low magnification image. Phalloidin fluorescence intensity in the alveoli was measured at the intersection of the lines. Intensities of 10 or more points per section were averaged and shown at the means ± SD of fold changes relative to controls, with sections from three individuals/group observed (*n* = 3); *P < 0.05, **P < 0.01 vs. control mice, as determined by Student's *t*‐test.

**Fig. 6:**
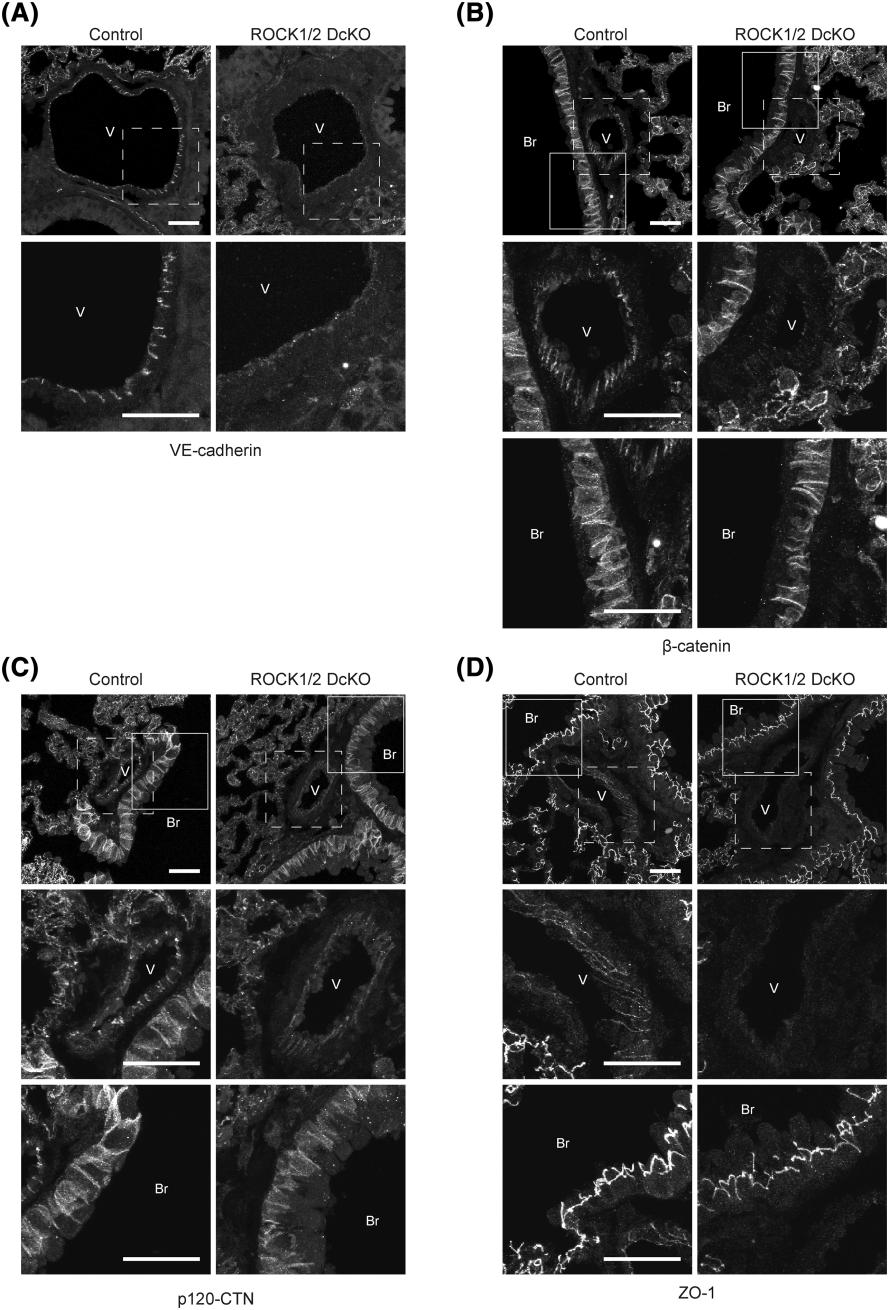
Decrease in localization of vascular endothelial (VE)‐cadherin, β‐catenin, p120‐catenin (p120‐CTN) and zonula occludens‐1 (ZO‐1) to lung endothelial cells in Rho‐associated coiled‐coil kinase 1 and Rho‐associated coiled‐coil kinase 2 double conditional knockout (ROCK1/2 DcKO) mice. (A) Upper panels: representative images of lung sections from lungs harvested from control and ROCK1/2 DcKO mice on Day 5 following the course of tamoxifen (TAM) treatment and stained with antibodies against VE‐cadherin. The lower panels contain enlarged images of the boxed areas in the upper panels. Images of entire tissue were acquired every 0.7 μm, and the overlay images are shown. Scale bars: 30 μm. (B–D) Upper panels: representative images of lung sections from lungs harvested from control and ROCK1/2 DcKO mice on Day 5 following the course of TAM treatment and stained with antibodies against β‐catenin (B), p120‐CTN (C), and ZO‐1 (D). The middle or lower panels contain enlarged images of the boxed areas (dotted or solid line) in the upper panels, respectively. Images of entire tissue were acquired every 0.7 μm, and the overlay images are shown. Scale bars: 30 μm. In panels A–D, V or Br indicates a pulmonary vessel or bronchus, respectively.

The maintenance of vascular permeability is linked to actin cytoskeleton remodeling [26]. We analyzed F‐actin levels in the lungs of ROCK1/2 DcKO mice through phalloidin staining (Fig. 5). On the last day of TAM treatment (Day 0) of the control and ROCK1/2 DcKO groups, there was no difference in the fluorescence intensity of phalloidin in lung sections. In contrast, phalloidin fluorescence was significantly decreased on Days 3 and 5 post‐TAM in ROCK1/2 DcKO mice compared to that in control mice (day 3: 0.68 ± 0.08‐fold, *P* < 0.05; day 5: 0.60 ± 0.08‐fold, *P* < 0.01; Fig. 5). Previous studies have shown that actin fibers serve as a scaffold for VE‐cadherin complexes [27]. The intracellular domain of VE‐cadherin binds to both β‐catenin and p120‐CTN. β‐catenin further interacts with the actin cytoskeleton, directly or indirectly [28, 29]. Therefore, we examined the localization of VE‐cadherin (Fig. 6A), β‐catenin (Fig. 6B), and p120‐CTN (Fig. 6C) in pulmonary vascular endothelial cells of ROCK1/2 DcKO and control mice by immunofluorescence staining. GSL I‐Isolectin B4 was used as a marker for vascular endothelial cells [30]. Fluorescent signals corresponding to VE‐cadherin were observed in pulmonary vascular endothelial cells of control mice. However, in ROCK1/2 DcKO mice on Day 5 post‐TAM, the intensity of the fluorescent signal in these cells was reduced; even among vessels with a detectable signal, none exhibited fluorescent signals throughout the vessel (Fig. 6A and Fig. S3). Similarly, fluorescent signals for β‐catenin and p120‐CTN were observed in pulmonary vascular endothelial cells from control mice but were attenuated in those from ROCK1/2 DcKO mice (Fig. 6B,C, Figs S4, and S5). Strong fluorescent signals corresponding to β‐catenin and p120‐CTN were observed in the bronchial epithelial cells of ROCK1/2 DcKO mice but were disorganized and irregular in length compared with those in control mice (Fig. 6B,C). The localization of ZO‐1, which components tight junction, was also observed. The fluorescent signals of ZO‐1 on vessels in ROCK1/2 DcKO mice tended to show a reduction compared with those in control mice on Day 5 post‐TAM (Fig. 6D and Fig. S6).

### Maintenance of the VE‐cadherin complex in vascular endothelial cells is mediated by ROCK signaling both *in vivo* and *in vitro*

**Fig. 7:**
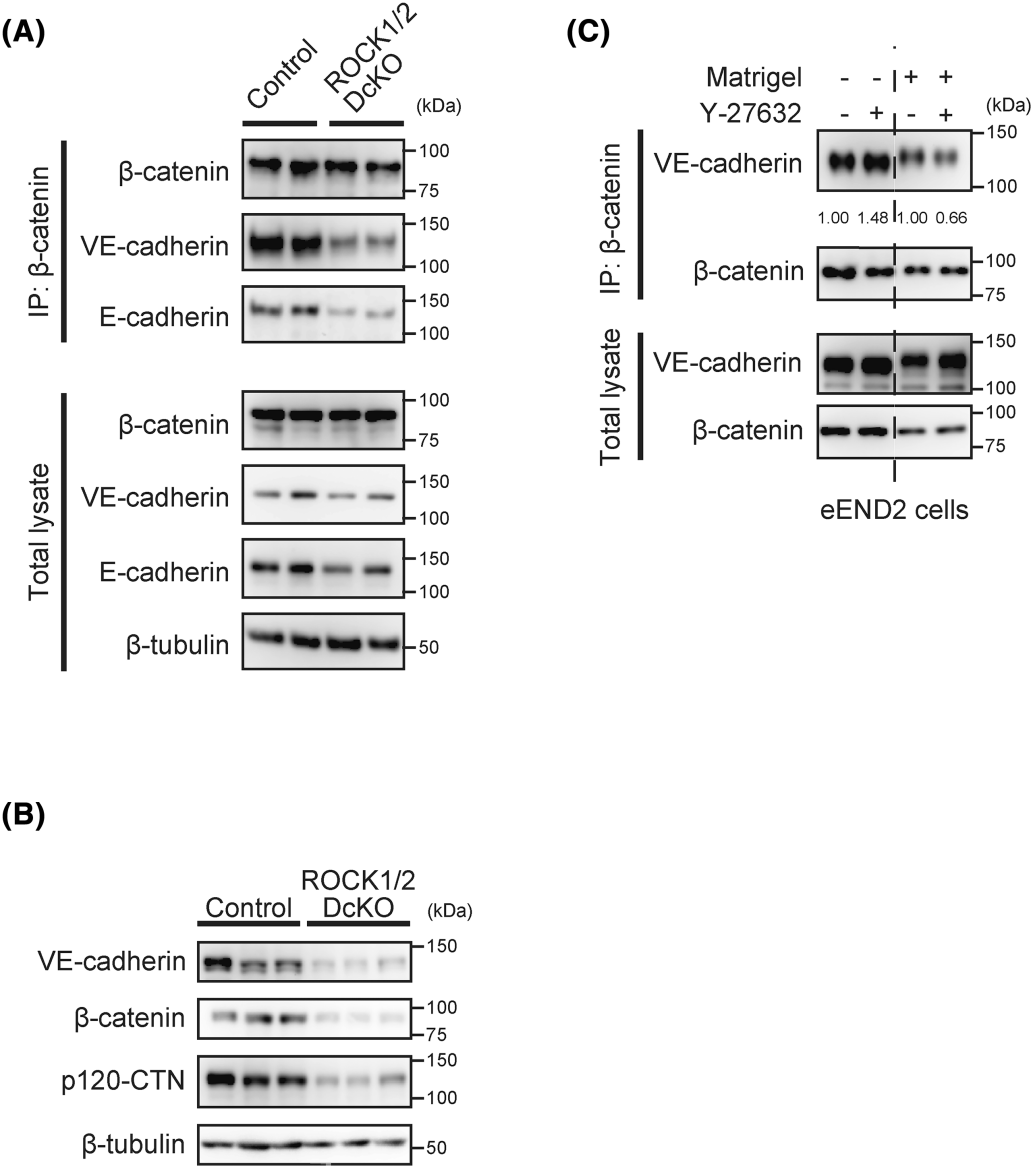
Suppression of vascular endothelial (VE)‐cadherin/β‐catenin complex formation caused by loss of Rho‐associated coiled‐coil kinase (ROCK) activity. (A, B) Immunocomplexes and total lung lysates from control and ROCK1/2 double conditional knockout (ROCK1/2 DcKO) mice on Days 5 or 7 following the tamoxifen (TAM) course were analyzed by coimmunoprecipitation (IP) and western blotting with the indicated antibodies. (A) Day 5 lung lysates: co‐IP with anti‐β‐catenin antibody and western blotting with the indicated antibodies. Each group contains protein obtained from two different individuals. (B) Day 7 lung lysates: western blotting with the indicated antibodies. Each group contains protein obtained from three different individuals. (C) eEND2 cells cultured on plastic dishes with or without Matrigel and treated with 15 μm Y‐27632 for 1 h, followed by co‐IP with anti‐β‐catenin antibody or western blotting of immunocomplexes and total eEND2 cell lysates with the indicated antibodies. The intensities of VE‐cadherin bands were corrected for the intensities of the corresponding β‐catenin bands. The ratios are presented as fold changes relative to dimethyl sulfoxide (DMSO) control‐treated cells.

To examine the effects of ROCK1/2 deficiency on VE‐cadherin/β‐catenin complex formation, we performed IP experiments with anti‐β‐catenin antibodies on total lung lysates from ROCK1/2 DcKO and control group mice (Fig. 7A). On Day 5 following the TAM course, VE‐cadherin and E‐cadherin co‐precipitated with β‐catenin in both groups; however, the amount of coprecipitation of VE‐cadherin and E‐cadherin was obviously reduced in ROCK1/2 DcKO mice. Next, the levels of each protein in lung on Days 5 (Fig. 7A) and 7 (Fig. 7B) post‐TAM were analyzed by western blotting. On Day 5, there was no difference in the lung levels of these proteins between the two groups, but by Day 7, VE‐cadherin, β‐catenin, and p120‐CTN levels were decreased in the lungs of ROCK1/2 DcKO mice compared with those in control mice. In contrast, there were no between‐group differences in the Day 7 levels of these proteins in the liver (Fig. S7). Finally, we examined the influence of ROCK inhibition on the amount of the VE‐cadherin/β‐catenin complex in cultured mouse vascular endothelial‐like eEND2 cells, which develop tubes on Matrigel. eEND2 cells seeded on plastic dishes with or without Matrigel coating were treated with 15 μM Y‐27632 for 1 h, and then subjected to IP using anti‐β‐catenin antibody (Fig. 7C). The amount of VE‐cadherin that co‐precipitated with β‐catenin increased by 1.48‐fold under Y‐27632 treatment in cEND2 cells cultured on plastic dishes. In contrast, Y‐27632 treatment decreased complex formation by 0.66‐fold in the tube‐forming eEND2 cells cultured on Matrigel. These findings suggested that ROCK proteins have a role in vascular maintenance, and that inhibiting or reducing ROCK activity suppresses VE‐cadherin/β‐catenin complex formation in endothelial cells both *in vitro* and *in vivo*.

## Discussion

In this study, we generated TAM‐inducible ROCK1 cKO, ROCK2 cKO, and ROCK1/2 DcKO mice and analyzed the body‐wide functions of ROCK1 and ROCK2 in adult mice. Single cKO mice showed no significant BP fluctuations and survived for nearly 2 weeks following TAM treatment. In contrast, ROCK1/2 DcKO mice showed reductions in SBP, MBP, and DBP at Day 5, and all had died by Day 11 post‐TAM. Lung hemorrhage and lung enlargement were observed in ROCK1/2 DcKO mice, resulting in increased pulmonary vascular permeability. On Day 3 post‐TAM, F‐actin content decreased in the lungs, and by Day 5, the levels of VE‐cadherin, β‐catenin, and p120‐CTN localized within cell–cell adhesion sites of pulmonary vascular endothelial cells were reduced in ROCK1/2 DcKO mice. Furthermore, VE‐cadherin/β‐catenin complexes were destabilized, resulting in decreased levels of each protein. These results suggested that, in the body, ROCK proteins are involved in the maintenance of lung functions through the regulation of cell adhesion mediated by VE‐cadherin/β‐catenin complexes.

Pharmacological inhibition by ROCK inhibitors has advanced analyses of the physiological functions of ROCKs in cultured cells and in the body [31]. In particular, animals treated with Y‐27632 show reduced BP, suggesting that ROCKs contribute to BP regulation [23, 32, 33]. Furthermore, the mechanism of BP regulation by ROCKs is reportedly mediated by increased calcium sensitivity [23]. ROCK2 inhibition by the ROCK2 selective inhibitor KD025 does not cause a large reduction in BP [34]. These findings are consistent with our results; that is, obvious decreases in SBP, MBP, and DBP were observed in ROCK1/2 DcKO mice, but not in ROCK1 or ROCK2 cKO mice. Thus, our findings support the coordinated involvement of ROCK1 and ROCK2 in BP regulation. Additionally, in a previous report, blood analyses of ROCK1/ROCK2 knockout mice have shown that several factors, such as glucose, triglyceride, albumin, and BUN, fluctuate [35], suggesting that the two ROCK isoforms also regulate functions in other organs in a coordinated manner. Furthermore, ROCK1 and ROCK2 are widely expressed in the body, but ROCK2 is particularly strongly expressed in some organs such as brain and skeletal muscle [36]. The transgenic mice generated in this study may be useful to analyze the cooperative or isoform‐specific functions of ROCKs *in vivo*.

Vascular permeability is reportedly multifaceted and regulated in multiple ways: first, by impaired adhesion of vascular endothelial cells themselves; and second, by factors such as inflammatory cytokines that impair vascular endothelial cell adhesion and blood retention through inflammation or vasodilation [24, 25, 37].

Regarding the first possibility, previous reports have shown that activation of Rho‐ROCK signaling leads to disruption of cell–cell adhesion through cell tension generated by actomyosin activation in cultured human umbilical vein endothelial cells (HUVEC) [38, 39]. Furthermore, ROCK inhibitors have been reported to suppress the increase in vascular permeability that occurs in response to infection or inflammatory agents *in vivo* [40, 41]. Thus, activation of ROCK is thought to be involved in the increase in vascular permeability. We had similar observations in this study, finding that ROCK inhibition by Y‐27632 stabilized the VE‐cadherin/β‐catenin complex in vascular endothelial cells cultured on plastic dishes. However, we also showed that VE‐cadherin/β‐catenin complex formation was attenuated in ROCK1/2 DcKO mice, and that this complex was destabilized by treatment with the ROCK inhibitor in vascular endothelial cells cultured on Matrigel. These results suggested that cell–cell adhesion is severely impaired by inactivation or deletion of ROCK genes. In a previous report, Yamamoto *et al*. [42] showed that the amount of immunoprecipitated p120 bound to VE‐cadherin from mouse lungs was reduced by treatment with the ROCK inhibitor H‐1152. The ROCK inhibitor Y‐27632 is reportedly ineffective against airway microvascular leakage induced by leukotriene D4 and histamine *in vivo* [43]. Sambandam *et al*. [35] reported that intestinal barrier function is impaired in ROCK1/2 DcKO mice. In this context, the authors emphasized the involvement of ROCK in the maintenance of stem cells, and that ROCK deficiency may additionally contribute to cell adhesion of the intestinal epithelial cells themselves. Taken together, these findings suggest that ROCK activity may contribute to the maintenance of barrier function *in vivo* through the regulation of cell adhesion complexes.

Regarding the second possibility, in this study we did not observe an obvious increase in various molecules that induce vascular permeability in ROCK1/2 DcKO mice. However, increases in IL‐6 and CCL2 indicated that inflammation was induced in our ROCK1/2 DcKO mice. Furthermore, BP was reduced in our ROCK1/2 DcKO mice. Because these changes contribute to the disruption of barrier function, the generation of endothelial cell‐specific ROCK1/2 knockout mice will be essential for the analysis of ROCK‐mediated regulation of barrier function in future studies.

As mentioned above, isoform‐nonselective ROCK inhibitors, namely Y‐27632, H‐1152, and fasudil, have been widely used in previous ROCK research. Recently, the ROCK2 inhibitor belumosudil (REZUROCK™) was approved in 2021 for the treatment of chronic graft‐versus‐host disease [44]. Other ROCK2 selective inhibitors have also been developed and are being used to explore isoform‐specific functions [34, 45]. Nonetheless, achieving tissue‐ and cell‐specific ROCK inhibition using these pharmacological approaches is difficult. We found that mice with deficiency in ROCK1 or ROCK2 alone, as well as tissue‐specific ROCK1 and ROCK2 deficiency, are viable, and will be valuable in the future ROCK research.

## Conflict of interest

The authors declare no conflict of interest.

### Peer review

The peer review history for this article is available at https://www.webofscience.com/api/gateway/wos/peer‐review/10.1002/2211‐5463.13802.

## Author contributions

TA, TT, and TI participated in research design. TA, TT, TS, RH, IA, FH, SN, and TI conducted experiments. HM and MI generated ROCK1^flox/flox^ mice. AT and TK generated ROCK2^flox/flox^ mice. KH, DT, and SN interpreted and discussed the results. TA, TT, and TI wrote the manuscript.

## Supporting information


**Fig. S1.** Schematic diagram of Rho‐associated coiled‐coil kinase 1 and Rho‐associated coiled‐coil kinase 2 genomic loci and targeting vectors.
**Fig. S2.** Effects of Rho‐associated coiled‐coil kinase deficiency on testes, livers, and kidneys.

**Fig. S3.** Reduced vascular endothelial‐cadherin localization in pulmonary endothelial cells from several individual Rho‐associated coiled‐coil kinase 1 and Rho‐associated coiled‐coil kinase 2 double conditional knockout (ROCK1/2 DcKO) mice.
**Fig. S4.** Reduced β‐catenin localization in pulmonary endothelial cells from several individual Rho‐associated coiled‐coil kinase 1 and Rho‐associated coiled‐coil kinase 2 double conditional knockout (ROCK1/2 DcKO) mice.
**Fig. S5.** Reduced localization of p120‐catenin (p120‐CTN) in pulmonary endothelial cells from several individual Rho‐associated coiled‐coil kinase 1 and Rho‐associated coiled‐coil kinase 2 double conditional knockout (ROCK1/2 DcKO) mice.
**Fig. S6.** Reduced localization of zonula occludens‐1 (ZO‐1) in pulmonary endothelial cells from Rho‐associated coiled‐coil kinase 1 and Rho‐associated coiled‐coil kinase 2 double conditional knockout (ROCK1/2 DcKO) mice.
**Fig. S7.** No difference in the levels of cell–cell adhesion proteins in livers between control and Rho‐associated coiled‐coil kinase 1 and Rho‐associated coiled‐coil kinase 2 double conditional knockout (ROCK1/2 DcKO) mice on Day 7 post‐tamoxifen (TAM).

**Table S1.** Primer sets used for genotyping.
**Table S2.** Primer sets used for reverse transcription quantitative polymerase chain reaction (RT‐qPCR) analysis.

## Data Availability

The data that support the findings of this study are contained within the article or Supporting information.
