# Supplementary material for: Conditional deficiency of Rho‐associated kinases disrupts endothelial cell junctions and impairs respiratory function in adult mice
Source: FEBS Open Bio. 2024 Apr 11;14(6):906–21. doi: 10.1002/2211-5463.13802 (PMC11148122; doi:10.1002/2211-5463.13802)
Supplement: Supplementary file 1 — Fig. S1. Schematic diagram of Rho‐associated coiled‐coil kinase 1 and Rho‐associated coiled‐coil kinase 2 genomic loci and targeting vectors. Fig. S2. Effects of Rho‐associated coiled‐coil kinase deficiency on testes, livers, and kidneys. [file FEB4-14-906-s003.pdf]

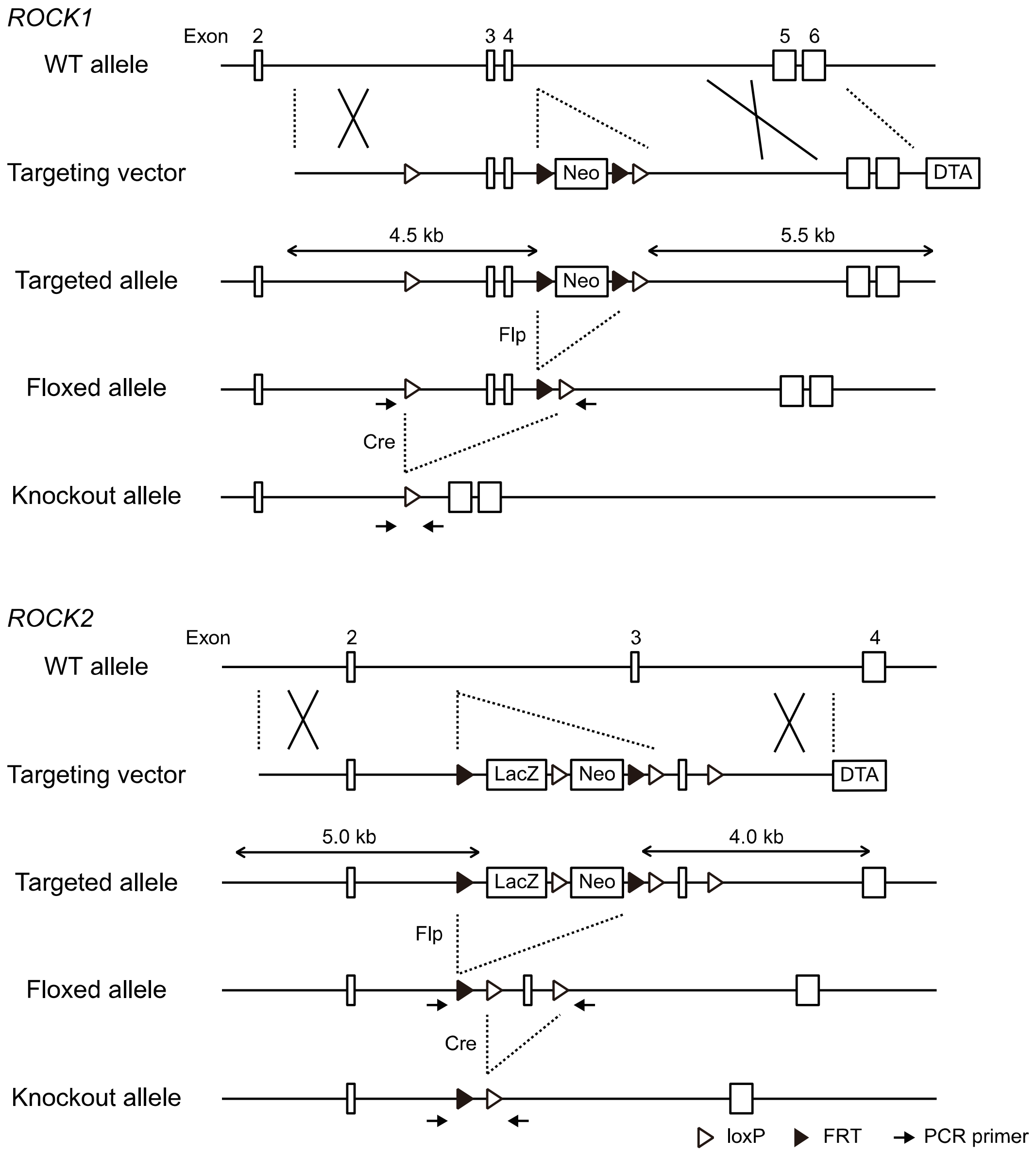

Fig. S1 Schematic diagram of Rho-associated coiled-coil kinase 1 (ROCK1) and Rho-associated coiled-coil kinase 2 (ROCK2) genomic loci and targeting vectors.
The wild-type (WT) allele lines show the respective ROCK1 and ROCK2 gene loci. Exons are indicated as white boxes and are numbered above. The targeting vectors for ROCK1 and ROCK2 were electroporated into embryonic stem (ES) cells. ES clones were microinjected into C57BL/6 blastocysts to generate chimeric mice. The chimeric mice were bred with WT C57BL/6 mice, generating offspring heterozygous for $ROCK1^{flox/+}$ or $ROCK2^{flox/+}$ alleles (Targeted allele). These heterozygous animals were crossed with mice expressing flippase (flp)-recombinase in the germline to delete the Neo cassette. The homozygous $ROCK1^{flox/flox}$ / $ROCK2^{flox/flox}$ mice that were obtained were bred with ubiquitin C (Ubc)-CreERT2 mice to generate Ubc-CreERT2;$ROCK1^{flox/flox}$/$ROCK2^{flox/flox}$ mice. The Floxed allele and Knockout allele lines show the respective genomic loci before and after tamoxifen (TAM)-induced deletion of ROCK1 and ROCK2.

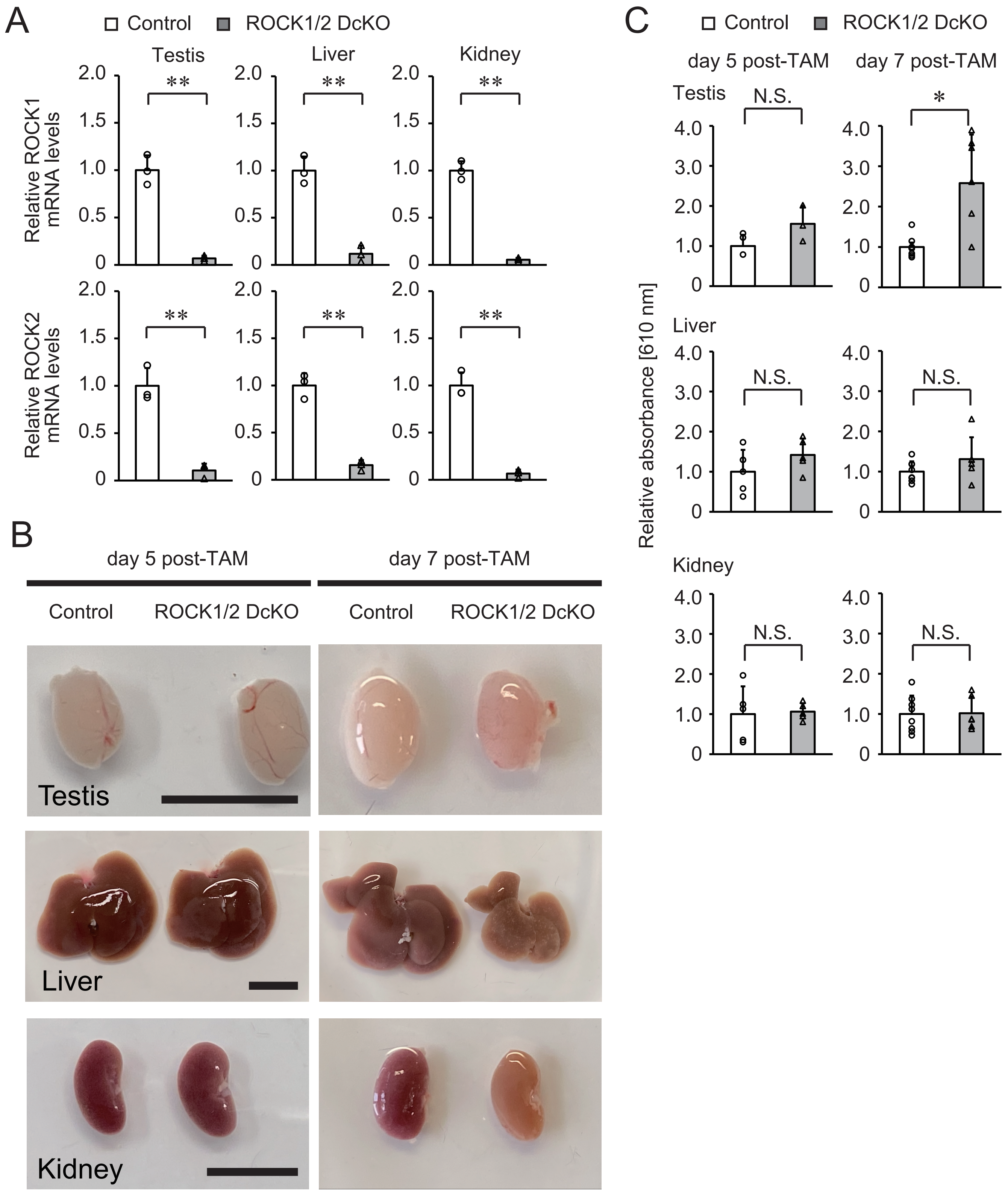

Fig. S2 Effects of Rho-associated coiled-coil kinase (ROCK) deficiency on testes, livers, and kidneys.
(A) Reverse transcription quantitative polymerase chain reaction (RT-qPCR) analysis of ROCK1 and ROCK2 mRNA expression in the testes (left panels), livers (middle panels), and kidneys (right panels) of control and ROCK1/2 double conditional knockout (ROCK1/2 DcKO) mice on day 5 post-tamoxifen (TAM). Data are shown as the means ± S.D. of fold changes relative to control mice (n = 3); **$p < 0.01$ vs. control mice, as determined by Student' s t test. (B) Representative photographs of testes (upper panels), livers (middle panels), and kidneys (lower panels) in control and ROCK1/2 DcKO mice on days 5 (left panels) and 7 (right panels) post-TAM. Scale bars: 10 mm. (C) Relative absorbance values of Evans blue dye extracted from testes (n = 3 per group on day 5 post-TAM; n = 8 for control mice and n = 6 for ROCK1/2 DcKO mice on day 7 post-TAM; upper panels), livers (n = 5 per group on day 5 post-TAM; n = 8 for control mice and n = 6 for ROCK1/2 DcKO mice on day 7 post-TAM; middle panels), and kidneys (n = 5 per group on day 5 post-TAM; n = 8 for control mice and n = 6 for ROCK1/2 DcKO mice on day 7 post-TAM; lower panels) of control and ROCK1/2 DcKO mice on days 5 (left panels) and 7 (right panels) post-TAM and measured at 610 nm. The absorbances were corrected for the weight of each organ and are shown as means ± S.D. of fold changes relative to control mice; *$p < 0.05$ vs. control mice, as determined by Student' s t test.
