# Supplementary material for: Conditional deficiency of Rho‐associated kinases disrupts endothelial cell junctions and impairs respiratory function in adult mice
Source: FEBS Open Bio. 2024 Apr 11;14(6):906–21. doi: 10.1002/2211-5463.13802 (PMC11148122; doi:10.1002/2211-5463.13802)
Supplement: Supplementary file 2 — Fig. S3. Reduced vascular endothelial‐cadherin localization in pulmonary endothelial cells from several individual Rho‐associated coiled‐coil kinase 1 and Rho‐associated coiled‐coil kinase 2 double conditional knockout (ROCK1/2 DcKO) mice. Fig. S4. Reduced β‐catenin localization in pulmonary endothelial cells from several individual Rho‐associated coiled‐coil kinase 1 and Rho‐associated coiled‐coil kinase 2 double conditional knockout (ROCK1/2 DcKO) mice. Fig. S5. Reduced localization of p120‐catenin (p120‐CTN) in pulmonary endothelial cells from several individual Rho‐associated coiled‐coil kinase 1 and Rho‐associated coiled‐coil kinase 2 double conditional knockout (ROCK1/2 DcKO) mice. Fig. S6. Reduced localization of zonula occludens‐1 (ZO‐1) in pulmonary endothelial cells from Rho‐associated coiled‐coil kinase 1 and Rho‐associated coiled‐coil kinase 2 double conditional knockout (ROCK1/2 DcKO) mice. Fig. S7. No difference in the levels of cell–cell adhesion proteins in livers between control and Rho‐associated coiled‐coil kinase 1 and Rho‐associated coiled‐coil kinase 2 double conditional knockout (ROCK1/2 DcKO) mice on Day 7 post‐tamoxifen (TAM). [file FEB4-14-906-s001.pdf]

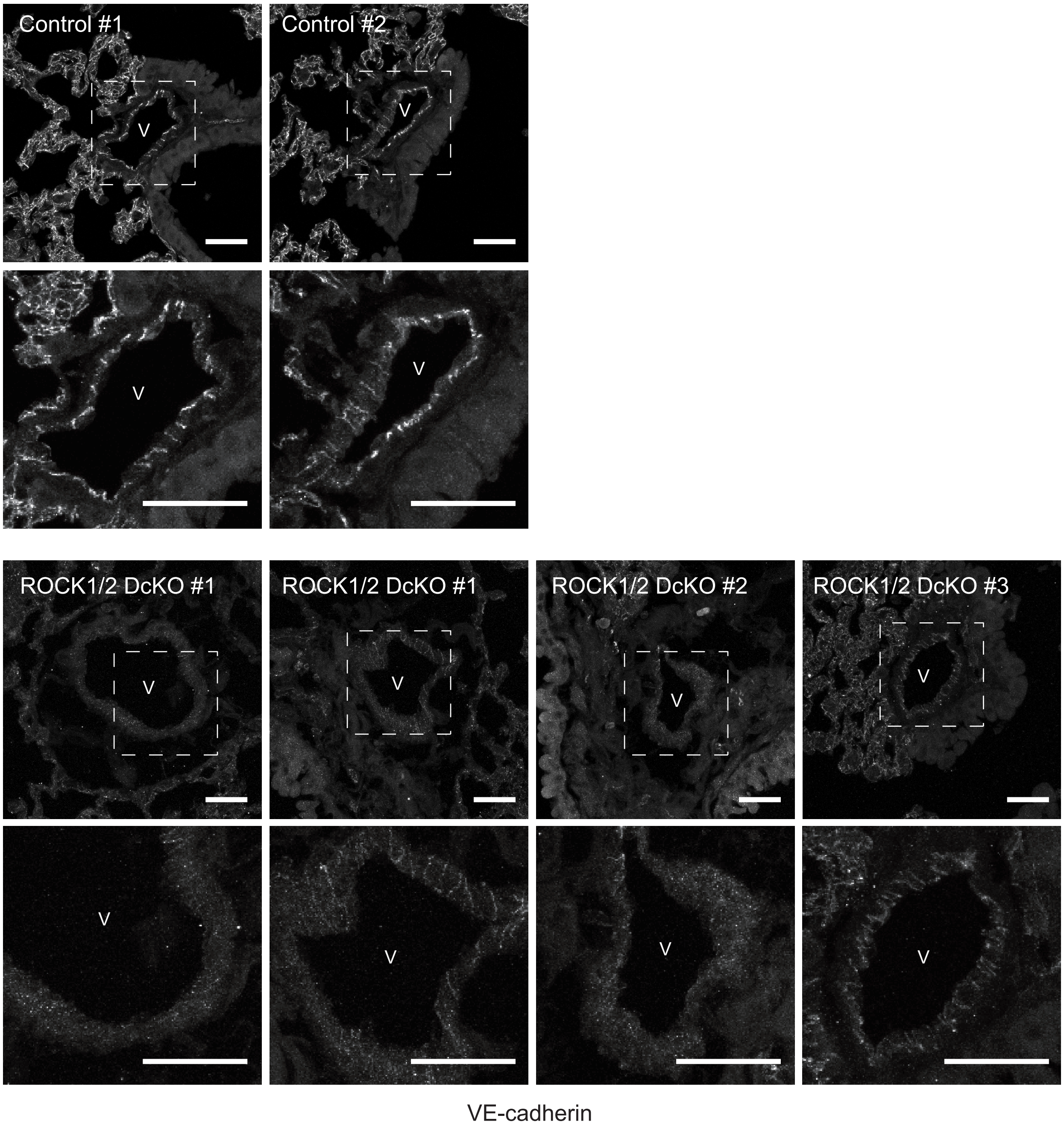

Fig. S3 Reduced vascular endothelial (VE)-cadherin localization in pulmonary endothelial cells from several individual Rho-associated coiled-coil kinase 1 and Rho-associated coiled-coil kinase 2 double conditional knockout (ROCK1/2 DcKO) mice.

Images of lung sections stained with anti-VE-cadherin antibody. Lungs were excised from control and ROCK1/2 DcKO mice on day 5 post-tamoxifen (TAM). The lower panels contain enlarged images of the boxed areas in the upper panels. Images of entire tissue were acquired every 0.7 μm, and the overlay images are shown. Scale bar: 30 μm. Individual mice in each group are indicated by numbers (#1-3). In panels, V indicates a pulmonary vessel.

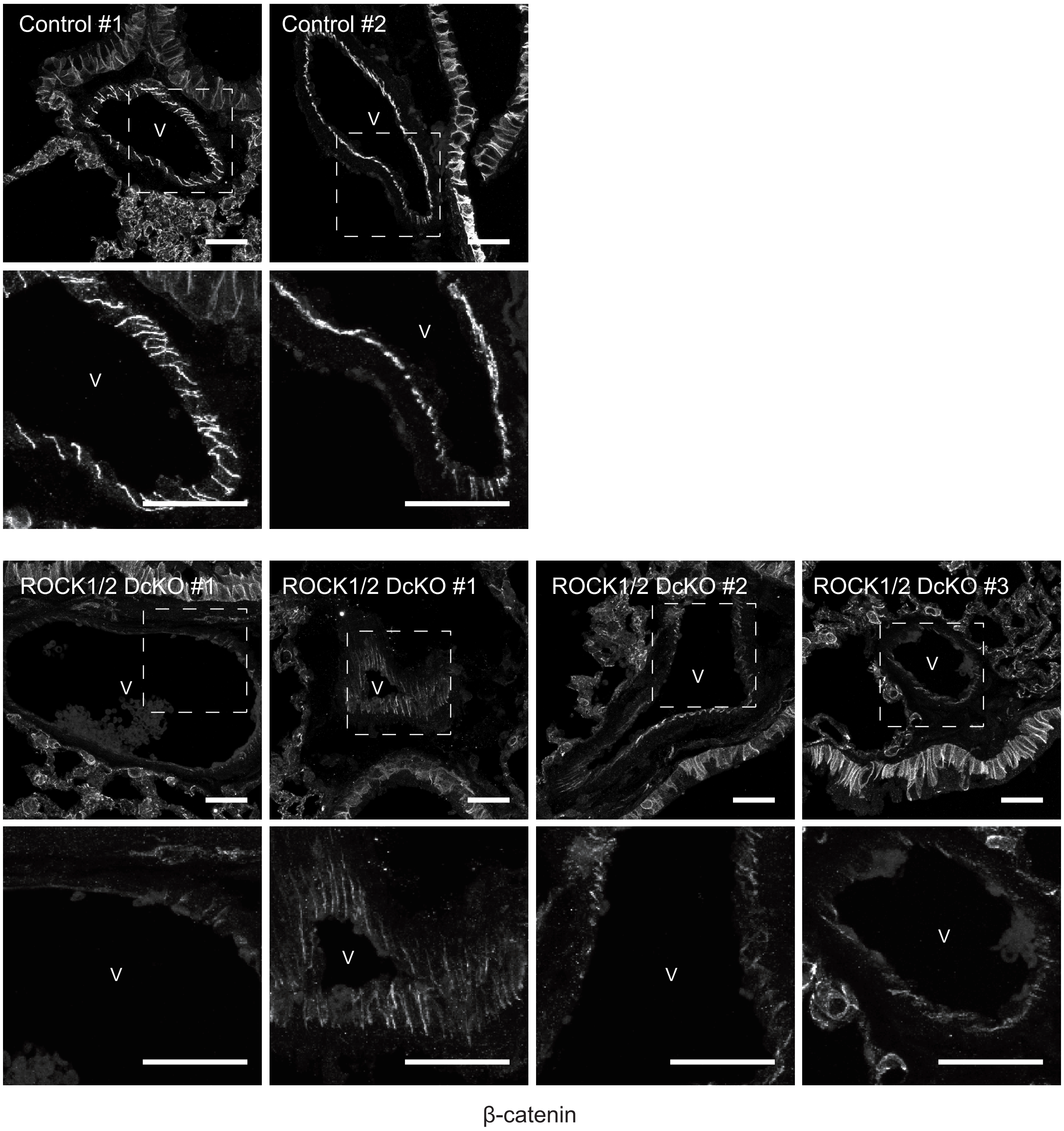

Fig. S4 Reduced β-catenin localization in pulmonary endothelial cells from several individual Rho-associated coiled-coil kinase 1 and Rho-associated coiled-coil kinase 2 double conditional knockout (ROCK1/2 DcKO) mice.

Images of lung sections stained with anti-β-catenin antibody. Lungs were excised from control and ROCK1/2 DcKO mice on day 5 post-tamoxifen (TAM). The lower panels contain enlarged images of the boxed areas in the upper panels. Images of entire tissue were acquired every 0.7 μm, and the overlay images are shown. Scale bar: 30 μm. Individual mice in each group are indicated by numbers (#1-3). In panels, V indicates pulmonary vessel.

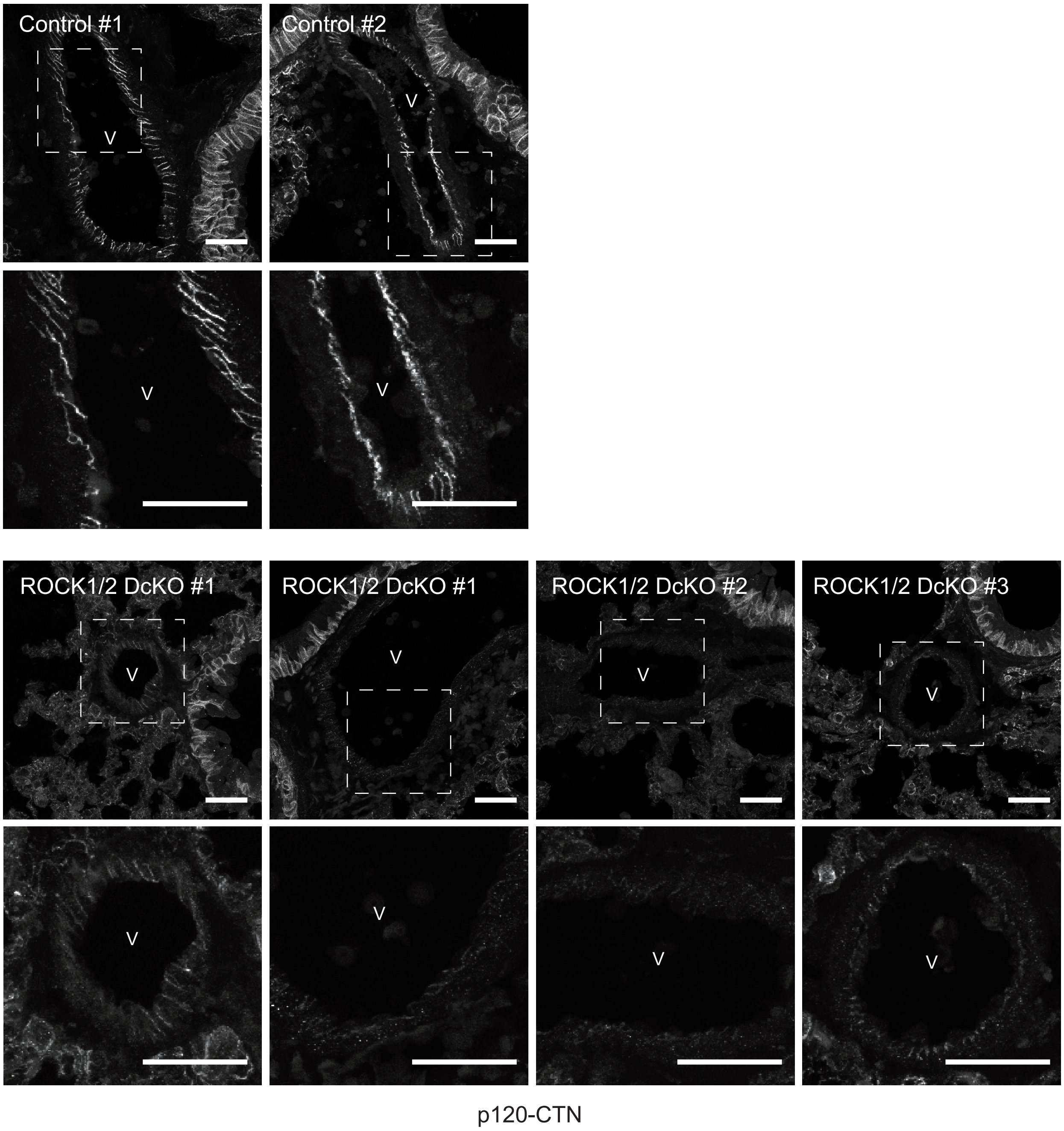

Fig. S5 Reduced localization of p120-catenin (p120-CTN) in pulmonary endothelial cells from several individual Rho-associated coiled-coil kinase 1 and Rho-associated coiled-coil kinase 2 double conditional knockout (ROCK1/2 DcKO) mice.

Images of lung sections stained with anti-p120-CTN antibody. Lungs were excised from control and ROCK1/2 DcKO mice on day 5 post-tamoxifen (TAM). The lower panels contain enlarged images of the boxed areas in the upper panels. Images of entire tissue were acquired every 0.7 μm, and the overlay images are shown. Scale bar: 30 μm. Individual mice in each group are indicated by numbers (#1-3). In panels, V indicates pulmonary vessel.

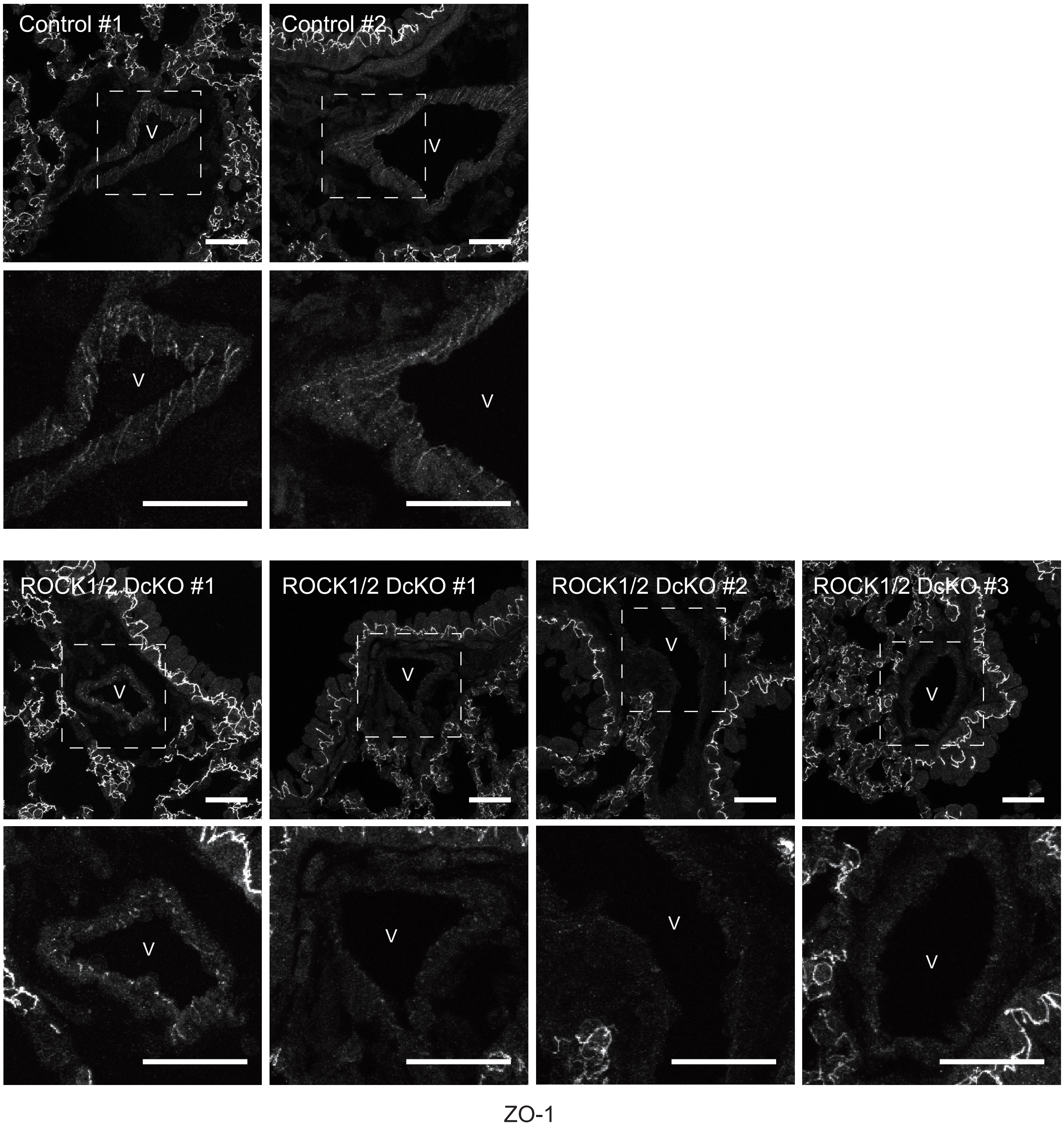

Fig. S6 Reduced localization of zonula occludens-1 (ZO-1) in pulmonary endothelial cells from Rho-associated coiled-coil kinase 1 and Rho-associated coiled-coil kinase 2 double conditional knockout (ROCK1/2 DcKO) mice.

Images of lung sections stained with anti-ZO-1 antibody. Lungs were excised from control and ROCK1/2 DcKO mice on day 5 post-tamoxifen (TAM). The lower panels contain enlarged images of the boxed areas in the upper panels. Images of entire tissue were acquired every 0.7 μm, and the overlay images are shown. Scale bar: 30 μm. Individual mice in each group are indicated by numbers (#1-3). In panels, V indicates a pulmonary vessel.

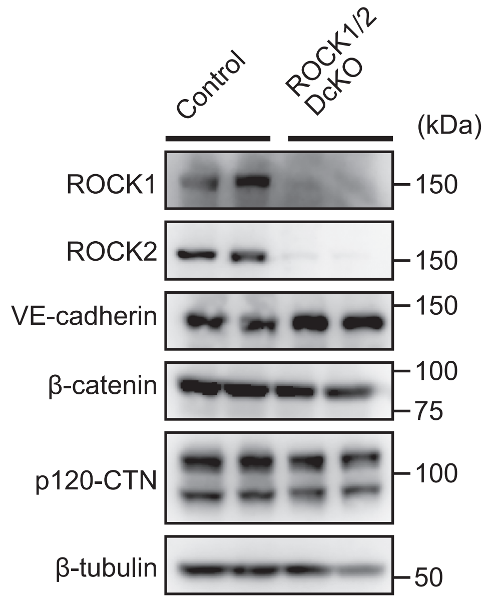

Fig. S7 No difference in the levels of cell-cell adhesion proteins in livers between control and Rho-associated coiled-coil kinase 1 and Rho-associated coiled-coil kinase 2 double conditional knockout (ROCK1/2 DcKO) mice on day 7 post-tamoxifen (TAM).
Total liver lysates from control and ROCK1/2 DcKO mice on day 7 following the TAM course were analyzed by western blotting with the indicated antibodies. Each group contains protein obtained from two different individuals.
