# Supplementary material for: Conditional deficiency of Rho‐associated kinases disrupts endothelial cell junctions and impairs respiratory function in adult mice
Source: FEBS Open Bio. 2024 Apr 11;14(6):906–21. doi: 10.1002/2211-5463.13802 (PMC11148122; doi:10.1002/2211-5463.13802)
Supplement: Supplementary file 3 — Table S1. Primer sets used for genotyping. Table S2. Primer sets used for reverse transcription quantitative polymerase chain reaction (RT‐qPCR) analysis. [file FEB4-14-906-s002.docx]

**Table S1. Primer sets used for genotyping.**

|  | **Forward (5’→3’)** | **Reverse (5’→3’)** |
| --- | --- | --- |
| ROCK1 5’ loxP | CAGCATTGCAGATGCCATCAG | CACCTGCTCACCAACACACC |
| ROCK1 3’ loxP | GTGTCTCTTACTGAAATCAGAAGGTTGAG | CAATTAGCCAGAAGTAGCACGTGC |
| ROCK2 5’ loxP | GCCTCGCACTTTCTGAGTCACTG | CAGAACCGCCTACTGCGACTATAGAG |
| ROCK2 3’ loxP | CTAAAATCCATTCTCAGCCATTGAGG | CAAGACCAGCTTCAGTCAGACCAG |
| *Cre* | GCATTACCGGTCGATGCAACGAGTGATGAG | GAGTGAACGAACCTGGTCGAAATCAGTGCG |

**Table S2. Primer sets used for RT-qPCR analysis.**

|  | **Forward (5’→3’)** | **Reverse (5’→3’)** |
| --- | --- | --- |
| ROCK1 | TGGCGTTTGCCAATAGTCCT | TTGCCCATTTTTCAGGCACG |
| ROCK2 | GTGGAGAGTCTGCTGGATGG | ACTGAACTTCACCAAAAGCACC |
| *Il-6* | ACTTCACAAGTCGGAGGCTT | GCAAGTGCATCATCGTTGTTC |
| *Ccl2* | TGCCCTAAGGTCTTCAGCAC | AAGGCATCACAGTCCGAGTC |
| *Tnf-α* | ATGGCCTCCCTCTCATCAGT | TTTGCTACGACGTGGGCTAC |
| *Il-1β* | GCCACCTTTTGACAGTGATGAG | GACAGCCCAGGTCAAAGGTT |
| *Nos2* | TCATGACATCGACCAGAAGC | GGACATCAAAGGTCTCACAG |
| *Nos3* | CGCAAGAGGAAGGAGTCTAGCA | TCGAGCAAAGGCACAGAAGTGG |
| *Vegfa* | GGAGATCCTTCGAGGAGCACTT | GGCGATTTAGCAGCAGATATAAGAA |
| *β-actin* | GGCTGTATCCCCTCCATCG | CCAGTTGGTAACAATGCCATGT |
